# Supplementary material for: missForestPredict—Missing data imputation for prediction settings
Source: PLoS One. 2025 Nov 7;20(11):e0334125. doi: 10.1371/journal.pone.0334125 (PMC12594382; doi:10.1371/journal.pone.0334125)
Supplement: S1 Appendix — (PDF) [file pone.0334125.s001.pdf]

Supporting Information for: missForestPredict – Missing data imputation for prediction settings

Supporting Information

RF imputation methods considered for comparison

Imputation methods based on random forests have been considered for comparison. The imputation methods in R have been included based on a cran repository search. From the python library scikit-learn we included IterativeImputer. Other python libraries like miceRanger or missingpy have been evaluated but excluded from the comparison as they are reproductions of the algorithms originally implemented in R. Table 1 presents an evaluation of the desired criteria for prediction settings for random forest imputation models. The code for the evaluating the capabilities of the R packages is available on github. The imputation methods that meet criteria 1 and 2 are compared to missForestPredict.

Based on the evaluation of the methods, bagging (in the caret R package and in the tidymodels framework), mice (method rf), miceRanger and the IterativeImputer from the python sci-kit learn library qualify for comparison (they meet the criteria (1) and (2)). Because the bagging implementation in caret and tidymodels is similar, with the difference that the tidymodels implementation can also impute categorical variables, we only include the tidymodels bagging imputation.

Variable transformations (dummy-coding)

To binarize categorical variables with K categories, we dummy code each category as $K-1$ binary variables, excluding the first category in alphabetical order. The same category is excluded on the training and test set. Imputation of these $K-1$ variables is done as if they were continuous. After continuous imputed values are "predicted" by the imputation model, they are backtransformed to categorical by treating the imputations as probabilities. The excluded category is calculated as $p_{iK} = 1 - \sum_{j=1}^{K-1} p_{ij}$, where p_{ij} is the prediction of class j for observation i and p_{iK} is the value of class K (last class) for observation i. To backtransform the $K-1$ variables to a categorical variable, the category corresponding to the variable with the highest predicted value is used. The choice of using $K-1$ categories and not K is made because an "imputation" model (using the complete dummy coded variables) could in theory perfectly learn a category in function of the other categories. As all categories will be missing when one category is missing, such a model is the worst possible imputation model. Let's take as example smoking status with values: Smoker, Not smoker, Ex-smoker. On the training data, the binary variables for the three categories will be either missing together or available together; an imputation model could learn that whenever both Smoker and Not smoker binary variables are 0, the value of Ex-smoker will be 1, without making use of other variables in the dataset, as the binary variables so created are treated as independent variables. At imputation time (imputing a new observation), Smoker, Not smoker, Ex-smoker will be missing together and the imputation model will rely on the initialized values in IterativeImputer and linear models.

Model building and tuning

Hyperparameter tuning for the random forest models is done using the tuneRanger R package (Probst, Wright, and Boulesteix 2018) and is based on the OOB error (logloss for binary outcomes and RMSE for continuous outcomes). The number of variables selected for each split (mtry) and the minimal node size (min.node.size) are the parameters tuned using sequential model-based optimization as described in Probst, Wright, and Boulesteix (2018) with 20 warmup iterations and 30 optimization iterations. The number of

Table 1: Comparison of capabilities of RF imputation methods

method	(1) Can impute a single new observation?	(2) Supports unsupervised imputation (= outcome not present)?	(3) Supports both continuous and categorical variables?	(4) Allows for new missingness patterns?
R - randomForest (rfImpute)	NO	NO	YES	NO
R - missRanger (missRanger)	NO	YES	YES	NO
R - mice (method="rf")	YES	YES	YES	NO
R - CALIBERrfimpute	NO	YES	YES	NO
R – imputeMissings impute(method = 'randomForest')	NO	YES	YES	NO
R - missForest	NO	YES	YES	NO
R - caret (bagImp)	YES	YES	NO	YES
R - tidymodels (bagging)	YES	YES	YES	YES
R - missForestPredict	YES	YES	YES	YES
R – miceRanger (miceRanger)	YES	YES	YES	YES
Python - scikit-learn 1.0 (IterativeImputer, estimator=RandomForestRegressor)	YES	YES	NO	YES

Table 2: Xgboost Hyperparameters

Hyperparameter	Value
Maximum number of boosting iterations	nrounds = c(100, 500)
Learning rate	eta = c(0.01, 0.1)
Maximum tree depth	max_depth = c(2, 4)
Minimum loss reduction to make a further partition on a leaf node of the tree	gamma = 0.01
Ratio defining the number of variables at each split	colsample_bytree = c(0.5, 1.0)
Minimum number of observations (sum of instance weight) needed to be in each node	min_child_weight = 0
Subsample ratio of the training observations	subsample = c(0.2, 0.5)

trees is set to 500. The parameter respect.unordered.factors is set to "order" as recommended in the package documentation; that means that categorical variables are ordered by their proportion falling in the second class (for binary classification) or by their mean response (for regression) and treated as ordered factors, as explained in Wright and König (2019).

The hyperparametes of the xgboost (XGB) model are tuned using 5-fold cross-validation using the caret package (Kuhn 2008) with the tuning grid presented in the Table 2.

For fitting the L2-regularized (logistic) regression models, the categorical variables are binarized (excluding one level) on both the training and test set and all variables are rescaled by subtracting the mean value of that variable and dividing by the standard deviation. The mean and standard deviation of each variable on the training set are stored and then applied on the test set. Five-fold cross validation is used on the training set for tuning the lambda regularization parameter using the glmnet R package (Friedman, Hastie, and Tibshirani 2010) with the default search grid of 100 lambda values.

For fitting the restricted cubic spline model the categorical variables are binarized on both the training and test set. The number of knots is set as 3 by default, and regression models are fitted with placing the interior knots at the 10%, 50%, and 90% quantiles of the continuous variables for the training dataset. Binary variables and ordinal variables which have too few levels to add 3 knots (that is: when any of the quantiles are equal) are kept in their original form. The rms R package is used to implement the cubic regression splines (Harrell Jr 2023).

Datasets description

This section provides additional information for each dataset used in comparisons: dataset description, preprocessing steps (whenever applicable), as well as the variable importance plot for the model built on the original data (on complete datasets with simulated missingness) or the variable importance plot for the model built on the data imputed using missForestPredict (on datasets with missing values). The variable importance is derived using the scaled permutation importance method implemented in ranger, as it is deemed to be less biased than other variable importance measures (Strobl et al. 2007). We provide the information of variable importance to offer the readers insight into whether variables with missing values are important in the model.

Diamonds

Data source: ggplot2 R package

The diamonds dataset is a Tiffany & Co's snapshot pricelist from 2017 and is part of the ggplot2 R package (Wickham 2016). The dataset contains attributes of almost 54,000 diamonds. The outcome is the price of diamonds (continuous). The dataset contains no missing values. The variable descriptions are available here: https://ggplot2.tidyverse.org/reference/diamonds.html. Descriptive statistics are available on kaggle: https://www.kaggle.com/datasets/shivam2503/diamonds.

Two diamonds have y dimension values of more than 30 mm, which is implausible for their carat value. These have been corrected by setting the y value to be the same as the x value. One diamond has the z value of more than 30 mm. This has been corrected to 60% of its length. Dimensionless diamonds (either of the x, y, z coordinates is zero), accounting for 20 observations in total, have also been considered faulty entries and have been corrected. Eight values of x and y equal to zero have been replaced with the mean value in the carat and cut group. Twenty values of z equal to zero have been replaced with $depth * (x + y)/2$ (using depth as percentage). One table value of 95 has been replaced with the mean in the carat and cut group. These preprocessing steps are inspired by a published notebook: https://rpubs.com/ankurmehta/diamond_outliers, which uses domain knowledge for outliers correction. Three ordinal variables (cut, color and clarity) have been transformed to continuous variables.

The resulting dataset has 53940 observations for 9 continuous variables. The outcome (price of the diamond) is continuous and is right skewed with expensive diamonds being less frequent.

Breast tumour

Data source: PMLB R package

The breast tumour dataset is part of the PMLB R package (Le, makeyourownmaker, and Moore 2020), a large collection of datasets for ML benchmarking maintained by the University of Pennsylvania (Olson et al. 2017) and contains patient (age, menopause) and tumour (nodes, malignant, etc.) variables. The patient cohort and the target variable are not documented. Descriptive statistics are available at https://epistasislab.github.io/pmlb/profile/1201_BNG_breastTumor.html.

Because the original dataset is very large (116640 observations), we have kept only half of the observations, using a random split. No preprocessing steps have been applied. Ordinal variable deg.mailg (degree of malignancy) is used as continuous. The dataset has 58320 observations on 4 continuous variables, 4 binary variables and one categorical variable with 3 categories (menopause). The outcome (target) seems to be multimodal.

Whitehall I

Data source: https://www.uniklinik-freiburg.de/imbi/stud-le/multivariable-model-building.html#c134656

The Whitehall I dataset contains data on British civil servants aged 40 - 64 collected in a cross-sectional cohort study between 1967 and 1969 (Royston, Ambler, and Sauerbrei 1999). Medical variables (smoking status, congestive heart disease, weight, height, vital signs and laboratory results: systolic and diastolic blood pressure, cholesterol) and socioeconomic data (job grade). The outcome is death within 10 years. Its

Table 3: Missing values per variable (Diabetes dataset)

Variable	No. missing	Percentage missing	Variable type
max_glu_serum	47169	94.95%	continuous
A1Cresult	41205	82.94%	continuous
medical_specialty	24234	48.78%	categorical, 4 categories
admission_source_id	3342	6.73%	categorical, 3 categories
discharge_disposition_id	2310	4.65%	categorical, 2 categories
race	1102	2.22%	categorical, 5 categories

original purpose was to investigate the association between socioeconomic factors (specifically job grade) and mortality after accounting for medical factors (Marmot, Shipley, and Rose 1984).

No preprocessing steps are applied. There are 17260 observations on 9 continuous variables and one binary variable (congestive heart disease). The job grade is an ordinal variable and is treated as continuous. There are 1670 civil servants (9.68%) who died within 10 years.

Diabetes

Data source: https://archive.ics.uci.edu/ml/datasets/Diabetes+130-US+hospitals+for+years+1999-2008

The diabetes dataset ia available on the UCI ML repository (Dua and Graff 2017) and contains 10 years (1999–2008) of clinical care data on diabetic patients from 130 hospitals in the United States extracted from the Health Facts database (an extraction from the Cerner Electronic Health Record System). Patient characteristics (gender, age group, etc.) and admission summaries (length of stay, number of procedures, number of medications, etc.) are available for 101766 patients. The outcome is hospital readmission within 30 days from discharge. Variables description and descriptive statistics are available at Strack et al. (2014). Because the original dataset is very large (101766 observations), we have kept only half of the observations, using a random split.

All entries with values: "?", "Unknown/Invalid", "None", "NULL", "Not Mapped" and "Not Available" on any variable are considered missing. Patient encounters resulting in death (expired) or discharge to palliative care (hospice) have been removed, as readmission prediction at the end of these encounters is not necessary. The primary diagnosis, coded in ICD-9 codes, has been collapsed to a categorical variable with 9 categories as described in Table 2 in the original study (Strack et al. 2014). Secondary diagnoses are not used. Sparse categorical variables (for which the most prevalent class had more than 99% of the values) have been removed (15 variables out of which 2 had a single value). Sparse Newborn and Trauma levels ($<$ 300 observations) for the admission type variable have been collapsed into Elective level. Discharge disposition has been collapsed to Home / Other and admission source into Emergency / Referral / Other as in the original study. The age variable is ordinal (grouped) and has been transformed to continuous. Eight other variables encoding medication changes (No, Down, Steady, Up) are ordinal in nature and have been transformed to continuous. Laboratory results max_glu_serum and A1Cresult with ordinal values have also been transformed to continuous. Variables payer_code and admission_type_id are not used. One observation with missing gender (0.002% of all observations) and 12 observations (0.02% of all observations) with missing diag_categ have been removed, leaving these variables as complete. The weight variable has been dropped because of extreme missingness ($>$ 95%).

The resulting dataset has 49679 observations on 27 variables out of which 6 contain missing values (Table 3). The resulting dataset has 27 variables; 19 continuous, 4 binary, 3 categorical variables with 3, 4 and respectively 5 categories and one categorical variable with 9 categories (diagnosis code). The outcome is readmission within 30 days and has a prevalence of 11.3% (5598 patients are readmitted within 30 days). This dataset is used in the comparison study as a dataset with missing values.

Further, the 6 remaining variables with missing values have been removed and the resulting dataset has 49679

Table 4: Missing values per variable (IST dataset)

Variable	No. missing	Percentage missing	Variable type
RDEF5	3939	20.29%	categorical, 2 categories
RDEF6	3446	17.75%	categorical, 2 categories
RDEF7	1590	8.19%	categorical, 2 categories
RDEF8	1248	6.43%	categorical, 2 categories
RATRIAL	984	5.07%	categorical, 2 categories
RASP3	984	5.07%	categorical, 2 categories
RDEF4	583	3%	categorical, 2 categories
RHEP24	344	1.77%	categorical, 2 categories
RDEF3	255	1.31%	categorical, 2 categories
RDEF1	246	1.27%	categorical, 2 categories
RDEF2	123	0.63%	categorical, 2 categories

on 21 variables: 17 continuous and 4 categorical (3 binary and one with 9 categories). Missing values are simulated on this complete dataset and performance is compared.

The International Stroke Trial (IST)

Data source: https://link.springer.com/article/10.1186/1745-6215-12-101 (Electronic supplementary material)

The International Stroke Trial (IST) contains data gathered during a randomised controlled trial conducted between 1991 and 1996 aiming to investigate the effects of aspirin and heparin administration on stroke outcomes (Sandercock, Niewada, and Członkowska 2011). Patients with a diagnosis of acute ischemic stroke are included in the trial and data are collected within 48 hours from the stroke onset. Data are subsequently collected at 14 days and 6 months with the primary outcome of the trial being "dead or dependent" at 6 month since onset. For the prediction model we will use the intermediary outcome of death at 14 days since the stroke onset, as it has less missing values for the outcome variable. Variables description is provided at Sandercock, Niewada, and Członkowska (2011).

Only variables collected at baseline (at randomisation time) are used, as well as the country code. 25 patients (0.13%) have missing or unknown outcome at 14 days. Although not the ideal approach for prediction settings, these patients have been removed for the purpose of the current study. 36 countries contributed to the data collection (categorical variable country has 36 levels), with some countries contributing with very few patients. Countries that contributed with less than 300 patients have been collapsed into the category "other". This leaves the country variable with 15 levels. All "C" ("can't assess") values for the deficit assessment variables have been considered missing.

The resulting dataset contains 19410 patients on 25 variables (5 continuous, 16 binary and 4 categorical with more than 2 categories: 3, 5, 7 and 15 categories); the largest number of categories is 15 for the country variable. The outcome is binary (death at 14 days) with prevalence of 10.5% (2034 patients die within 14 days).

COVID-19 Testing

Data source: medicaldata R package

The COVID-19 dataset is part of the medicaldata R package (Higgins 2021) and contains data concerning testing for SARS-CoV2 via PCR and additional patient and test settings information. A description of the dataset is available here: https://htmlpreview.github.io/?https://github.com/higgi13425/medicaldata/blob/master/man/description_docs/covid_desc.html. We hypothesise that predicting the covid test result at swab collection time could help with prioritization of samples in the lab (giving higher priority to samples at

Table 5: Missing values per variable (COVID-19 dataset)

Variable	No. missing	Percentage missing	Variable type
payor_group	7087	45.65%	categorical, 4 categories
patient_class	7077	45.59%	categorical, 6 categories
demo_group	1	0.01%	categorical, 3 categories

higher risk to turn out positive). The outcome is the covid test result (positive, negative or invalid). We collapse positive and invalid in one category as we consider that invalid tests need to be reprioritised quickly.

The covid tests occurred in 88 named clinics. All clinics with less than 300 observations have been collapsed to a single category (other), leaving the clinic name variable with 4 categories. Three low prevalence categories (less than 300 observations) for the variable patient class have been collapsed in the inpatient class; sparse categories for variable payor group have been collapsed into the "other" category; "misc adult" and "other adult" have been merged into adult category for variable demographic group. Variables measured after the swab collection time are discarded.

The resulting dataset has 15524 observations on 6 variables: 2 binary variables and 4 categorical variables with 3, 4, 4 and respectively 6 categories. The outcome (SARS-CoV2 PCR test result) is encountered for 1166 patients (7.5% of the total number of patients). The dataset has around 45% missing values for payor group and patient class and 1 single missing value for the demographic group (Table 5)

CRASH 2

Data source: https://biostat.app.vumc.org/wiki/Main/DataSets

The CRASH-2 dataset contains information gathered during a randomised controlled trial in 274 hospitals in 40 countries with the objective of studying the effects of administration of tranexamic acid among trauma patients (Williams-Johnson et al. 2010; Roberts et al. 2013). The primary outcome was death in hospital within 4 weeks of injury. Secondary outcomes (cause of death) are available but discarded for the current prediction purposes. Variables description can be found here: https://biostat.app.vumc.org/wiki/pub/Main/DataSets/Ccrash2.html.

Six sparse binary variables (less than 300 in a category level) have been removed. Telephone source has been collapsed with paper source because of sparseness. The dataset has 20207 observations on 27 variables: 15 continuous, 11 binary and 1 categorical variables with 3 categories. The outcome is death within 14 days and occurs for 3076 patients (15.2%). The dataset has around 50% missing values for 4 blood transfusion variables. Other 21 variables have low missingness rates of less than 4%. (See Table 6)

Complete cases

The number of complete cases on each dataset (after the previously mentioned deletions) are presented in Table 7.

OOB errors

OOB errors on simulated datasets

The OOB errors and the deviation of the imputed value from the true value on the test sets (NMSE) for simulated datasets with simulated missingness (amputation) are presented in Figures 1, 3, 2 and 4. To facilitate visualization, only four noise variables are presneted. Full results are available at: https://sibip.shinyapps.io/Results_imputation_methods/

Table 6: Missing values per variable (CRASH 2 dataset)

Variable	No. missing	Percentage missing	Variable type
nplasma	9964	49.31%	continuous
nplatelets	9964	49.31%	continuous
ncryo	9964	49.31%	continuous
ncell	9963	49.3%	continuous
gcsverbal	735	3.64%	continuous
gcseye	732	3.62%	continuous
gcsmotor	732	3.62%	continuous
cc	611	3.02%	continuous
bvii	374	1.85%	categorical, 2 categories
sbp	320	1.58%	continuous
rr	191	0.95%	continuous
ndaysicu	182	0.9%	continuous
hr	137	0.68%	continuous
bheadinj	80	0.4%	categorical, 2 categories
bneuro	80	0.4%	categorical, 2 categories
bchest	80	0.4%	categorical, 2 categories
babdomen	80	0.4%	categorical, 2 categories
bpelvis	80	0.4%	categorical, 2 categories
bbleed	80	0.4%	categorical, 2 categories
bmaint	80	0.4%	categorical, 2 categories
btransf	80	0.4%	categorical, 2 categories
gcs	23	0.11%	continuous
injurytime	11	0.05%	continuous
age	4	0.02%	continuous
sex	1	0%	categorical, 2 categories

Table 7: Complete cases (mean over all train/test splits) for datasets with simulated missingness (amputation)

dataset	No. (proportion) complete cases - train	No. (proportion) complete cases - test
breast tumor	1564.76 (0.04)	790.6 (0.04)
CRASH 2 - MV	6102.65 (0.45)	3048.35 (0.45)
diabetes - MV	45.5 (0)	22.5 (0)
diamonds	1453.03 (0.04)	722.55 (0.04)
diabetes	19.33 (0)	9.47 (0)
covid - MV	5626.57 (0.54)	2810.43 (0.54)
IST - MV	8719.69 (0.67)	4364.31 (0.67)
sim_75_1 - MAR_2	1303.23 (0.49)	652.22 (0.49)
sim_75_1 - MAR_2_out	1322.58 (0.5)	660.65 (0.5)
sim_75_1 - MAR_circ	656.81 (0.25)	331.04 (0.25)
sim_75_1 - MAR_circ_out	683.83 (0.26)	343.14 (0.26)
sim_75_1 - MCAR	639.29 (0.24)	321.44 (0.24)
sim_75_1 - MNAR	656.42 (0.25)	326.42 (0.25)
sim_75_1_noise - MAR_2	18.33 (0.01)	9.19 (0.01)
sim_75_1_noise - MAR_2_out	18.11 (0.01)	9.44 (0.01)
sim_75_1_noise - MAR_circ	9.22 (0)	4.3 (0)
sim_75_1_noise - MAR_circ_out	9.63 (0)	4.72 (0)
sim_75_1_noise - MCAR	8.61 (0)	4.57 (0)
sim_75_1_noise - MNAR	9.04 (0)	4.49 (0)
sim_75_7 - MAR_2	1360.65 (0.51)	680.94 (0.51)
sim_75_7 - MAR_2_out	1320.29 (0.5)	659.59 (0.5)
sim_75_7 - MAR_circ	798.72 (0.31)	400.73 (0.31)
sim_75_7 - MAR_circ_out	680.31 (0.26)	343.64 (0.26)
sim_75_7 - MCAR	639.29 (0.24)	321.44 (0.24)
sim_75_7 - MNAR	799.19 (0.31)	400.95 (0.31)
sim_75_7_noise - MAR_2	19.25 (0.01)	9.73 (0.01)
sim_75_7_noise - MAR_2_out	18.24 (0.01)	9.41 (0.01)
sim_75_7_noise - MAR_circ	11.42 (0)	5.4 (0)
sim_75_7_noise - MAR_circ_out	9.17 (0)	4.92 (0)
sim_75_7_noise - MCAR	8.61 (0)	4.57 (0)
sim_75_7_noise - MNAR	11.4 (0)	5.14 (0)
sim_90_1 - MAR_2	1315.21 (0.49)	657.24 (0.49)
sim_90_1 - MAR_2_out	1302.25 (0.49)	653.5 (0.49)
sim_90_1 - MAR_circ	661.44 (0.25)	331.83 (0.25)
sim_90_1 - MAR_circ_out	659.92 (0.25)	329.96 (0.25)
sim_90_1 - MCAR	639.29 (0.24)	321.44 (0.24)
sim_90_1 - MNAR	661 (0.25)	330.72 (0.25)
sim_90_1_noise - MAR_2	17.83 (0.01)	9.59 (0.01)
sim_90_1_noise - MAR_2_out	17.82 (0.01)	9.21 (0.01)
sim_90_1_noise - MAR_circ	9.3 (0)	4.16 (0)
sim_90_1_noise - MAR_circ_out	9.38 (0)	4.56 (0)
sim_90_1_noise - MCAR	8.61 (0)	4.57 (0)
sim_90_1_noise - MNAR	8.89 (0)	4.36 (0)
sim_90_7 - MAR_2	1359.46 (0.51)	678.49 (0.51)
sim_90_7 - MAR_2_out	1289.46 (0.48)	645.91 (0.48)
sim_90_7 - MAR_circ	797.03 (0.31)	398.56 (0.31)
sim_90_7 - MAR_circ_out	646.46 (0.24)	324.95 (0.24)
sim_90_7 - MCAR	639.29 (0.24)	321.44 (0.24)
sim_90_7 - MNAR	795.31 (0.3)	398.79 (0.3)
sim_90_7_noise - MAR_2	18.47 (0.01)	9.53 (0.01)
sim_90_7_noise - MAR_2_out	17.36 (0.01)	9.26 (0.01)
sim_90_7_noise - MAR_circ	10.15 (0)	5.32 (0)
sim_90_7_noise - MAR_circ_out	8.85 (0)	4.59 (0)
sim_90_7_noise - MCAR	8.61 (0)	4.57 (0)
sim_90_7_noise - MNAR	11.19 (0)	5.56 (0)
whitehall1	326.95 (0.03)	163.95 (0.03)

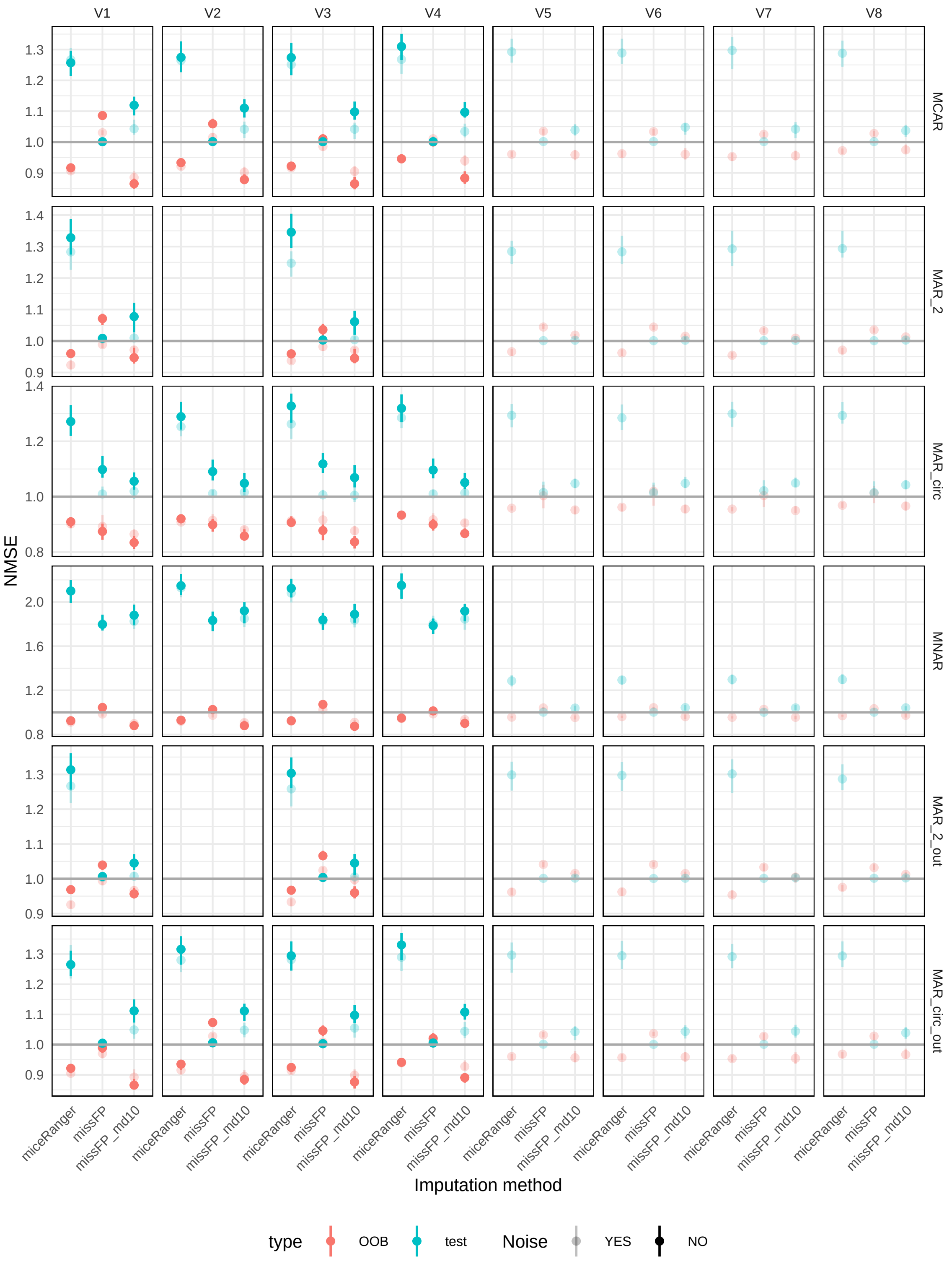

Figure 1: NMSE for continuous variables (OOB vs. test set) on simulated datasets: low correlation (0.1) and low AUROC (0.75)

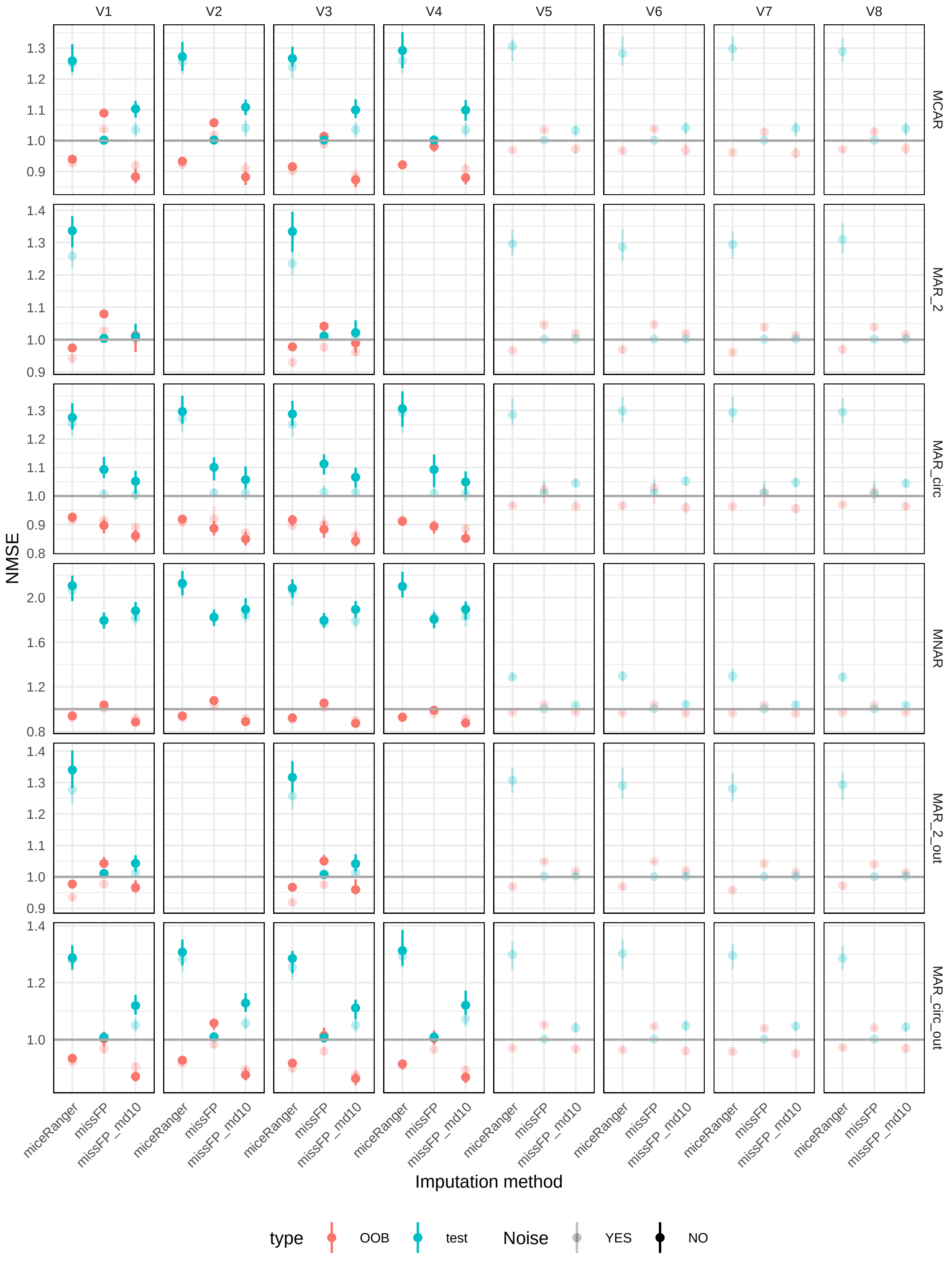

Figure 2: NMSE for continuous variables (OOB vs. test set) on simulated datasets: low correlation (0.1) and high AUROC (0.9)

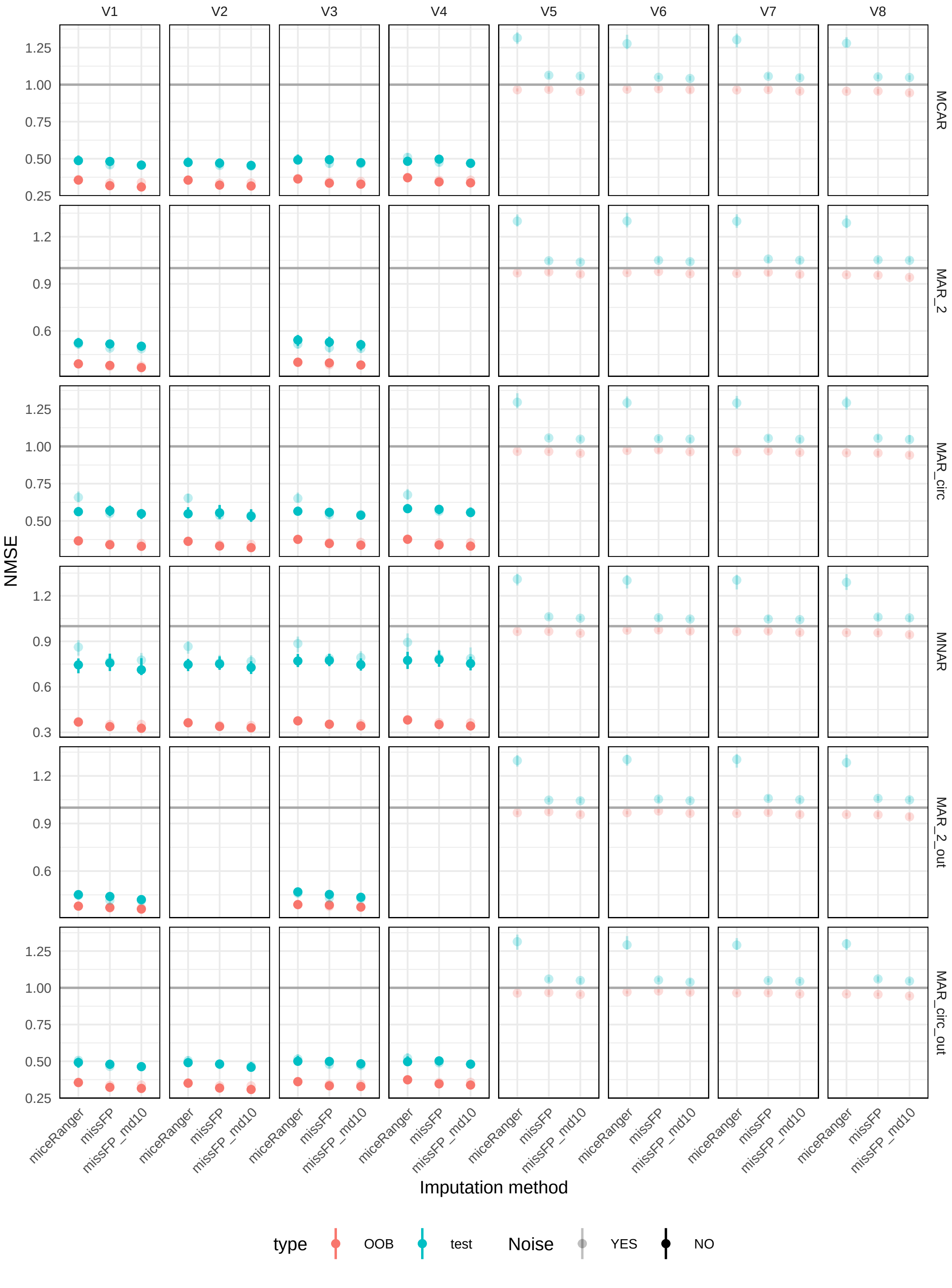

Figure 3: NMSE for continuous variables (OOB vs. test set) on simulated datasets: high correlation (0.7) and low AUROC (0.75)

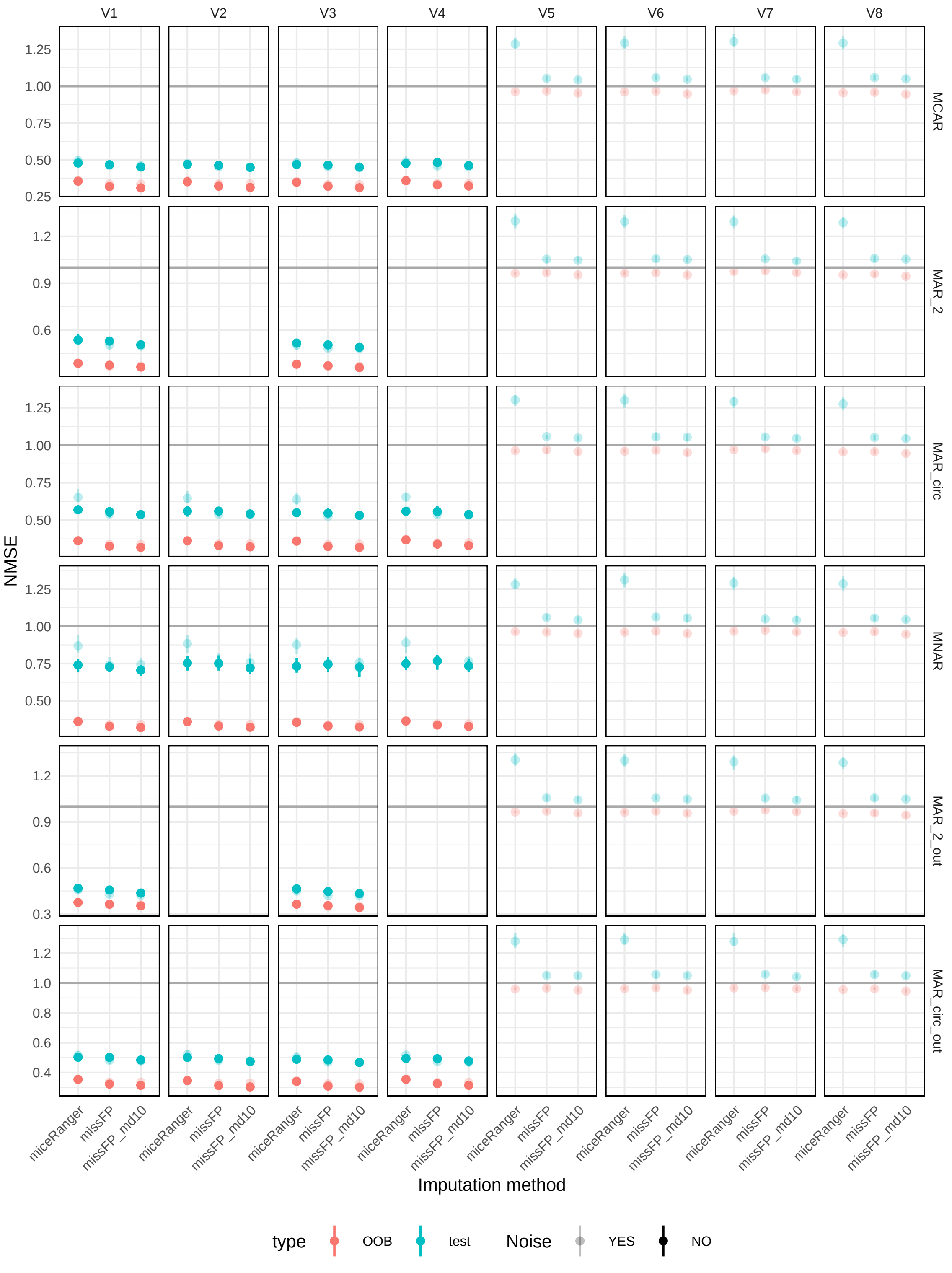

Figure 4: NMSE for continuous variables (OOB vs. test set) on simulated datasets: high correlation (0.7) high AUROC (0.9)

OOB errors on real datasets with simulated missingness

The OOB errors and the deviation of the imputed value from the true value on the test sets (NMSE for continuous variables and MER for categorical variables) are presented in Figure 5. To facilitate visualization, the top eight most important variables for each dataset are selected; the ranking is done using the variable importance of the random forest model built on the original dataset (with no missing values). Full results are available at: https://sibip.shinyapps.io/Results_imputation_methods/

OOB errors on real datasets with missing values

The OOB errors on the test sets (NMSE for continuous variables and MER for categorical variables) are presented in Figure 6. The true values on the test set are unknown. To facilitate visualization, the top eight most important variables for each dataset are selected; the ranking is done using the variable importance of the random forest model built on the missForestPredict imputed datatset. For the diabetes dataset, although there are more variables with missing values, only one (discharge_disposition_id) is in the top eight important variables. Full results are available at: https://sibip.shinyapps.io/Results_imputation_methods/

Iterations until convergence for missForestPredict

The number of iterations until the missForestPredict algorithm convergence is presented in Figure 7 for simulated datasets, Figure 8 for real datasets with simulated MCAR missingness and in Figure 9 for the real datasets with missing values.

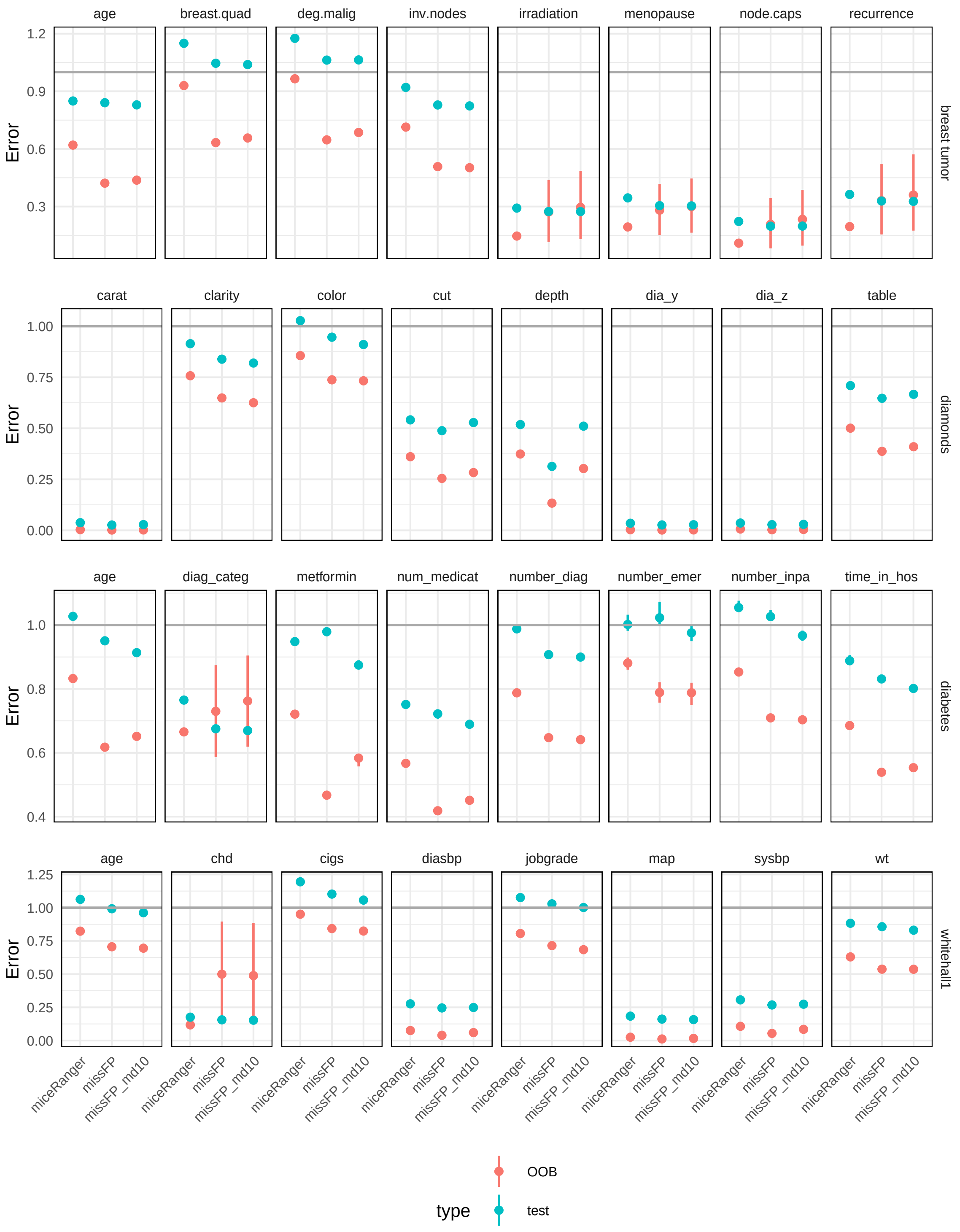

Figure 5: OOB and test set errors. For continuous variables the y-axis error is NMSE. For categorical variables the y-axis error is MER. The categorical variables are: diag_categ (diabetes dataset), irradiation, menopause, node.caps, recurrence (breast tumor dataset); chd (whitehall1 dataset). All other variables are continuous. To facilitate visualization, the top eight most important variables for each dataset are selected; the ranking is done using the variable importance of the random forest model built on the original dataset (with no missing values).

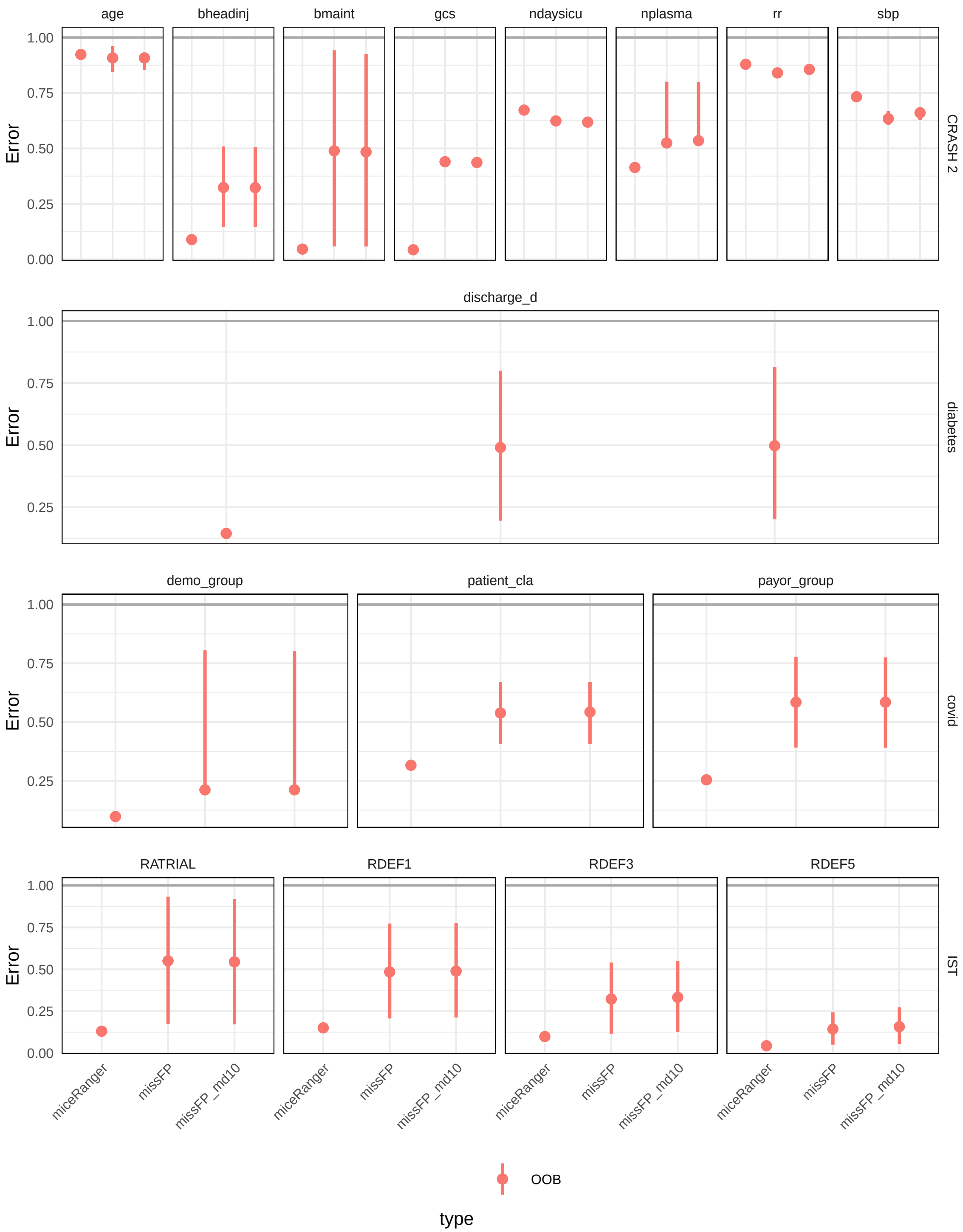

Figure 6: OOB errors. For continuous variables the y-axis error is NMSE. For categorical variables the y-axis error is MER. The categorical variables are: bheadinj, bmaint (CRASH-2), discharge_disposition_id (diabetes), all variables on the covid and IST datasets. All other variables are continuous. To facilitate visualization, the top eight most important variables for each dataset are selected; the ranking is done using the variable importance of the random forest model built on the original dataset (with no missing values)

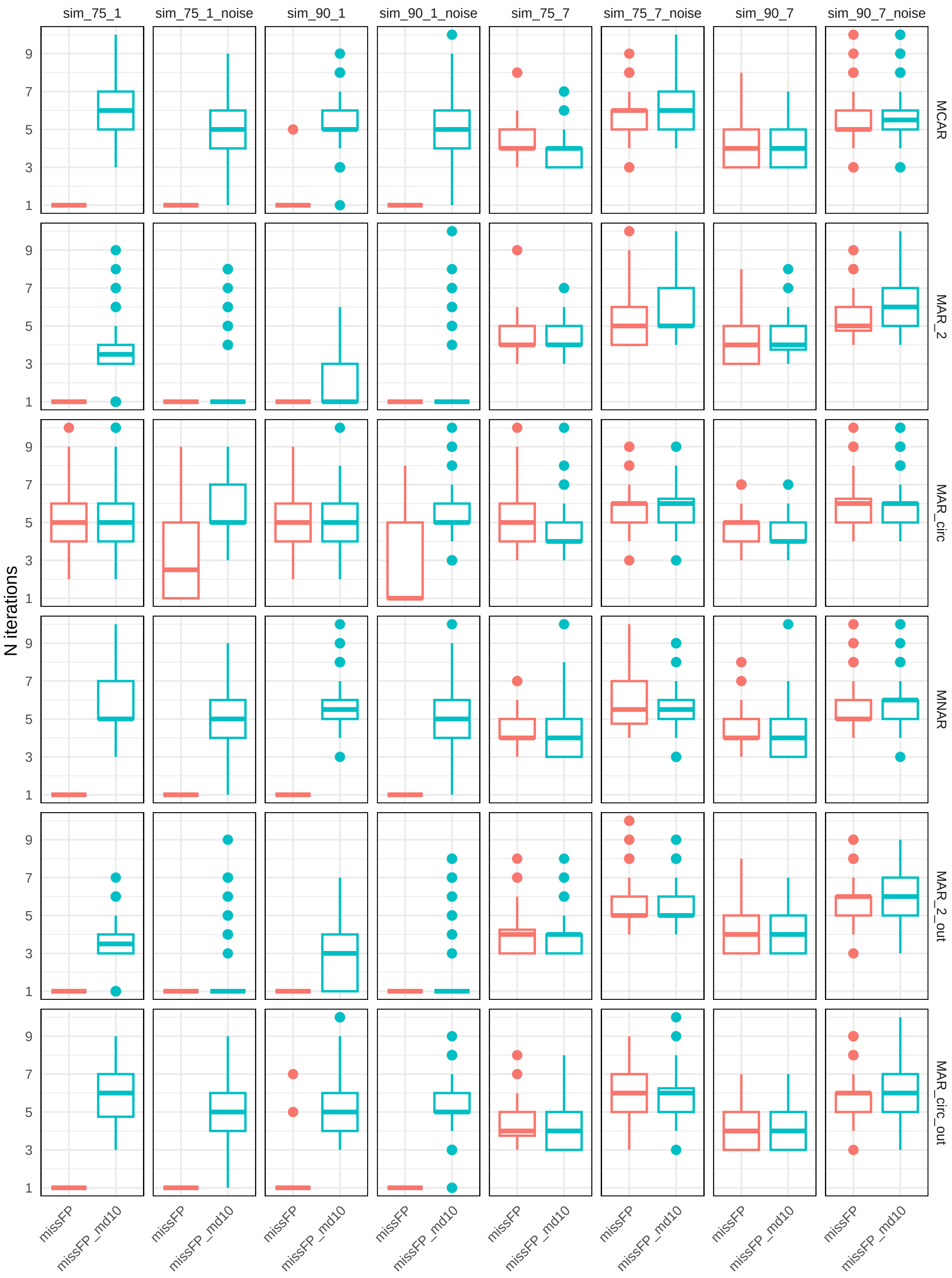

Figure 7: Number of iterations until convergence for missForestPredict with deep vs. shallow trees (simulated datasets)

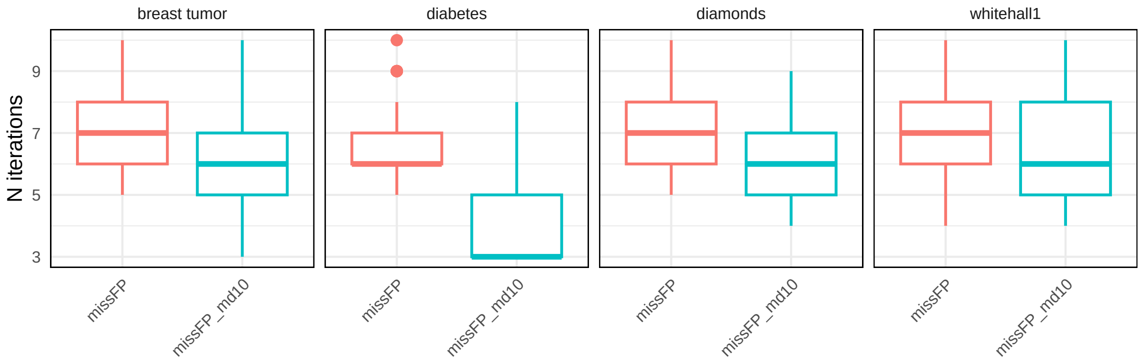

Figure 8: Number of iterations until convergence for missForestPredict with deep vs. shallow trees (real datasets with simulated MCAR missingness)

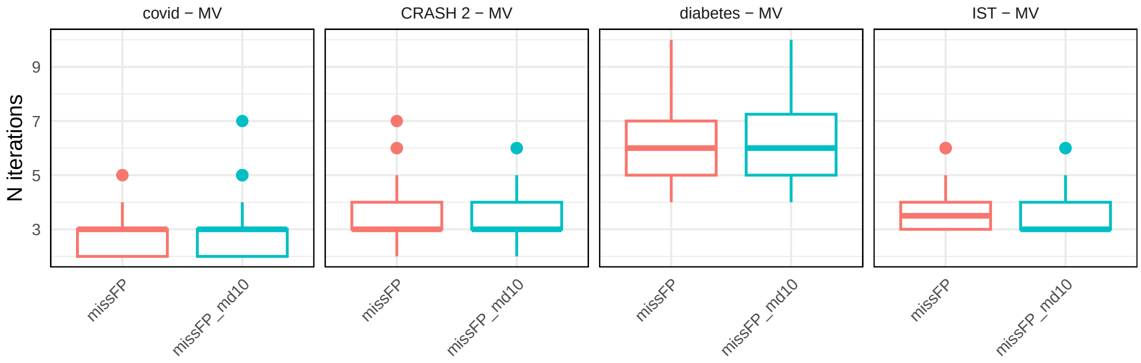

Figure 9: Number of iterations until convergence for missForestPredict with deep vs. shallow trees (real datasets with missing values)

Distribution comparison on real datasets with missing values

Covid dataset

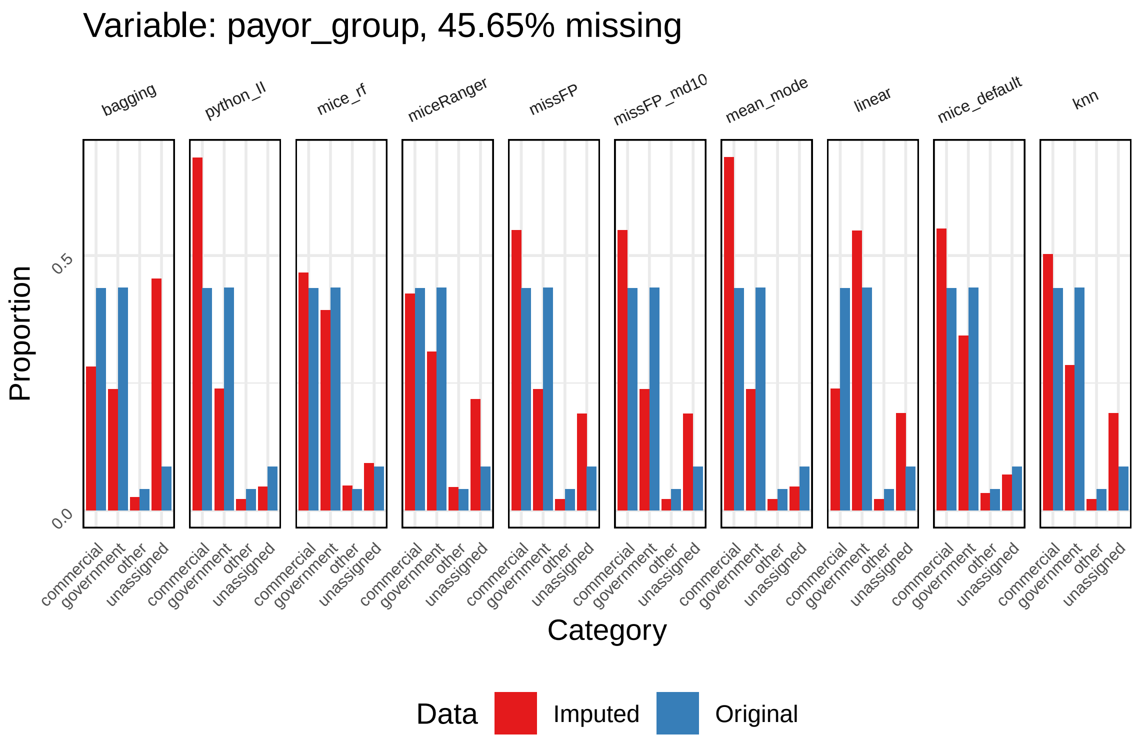

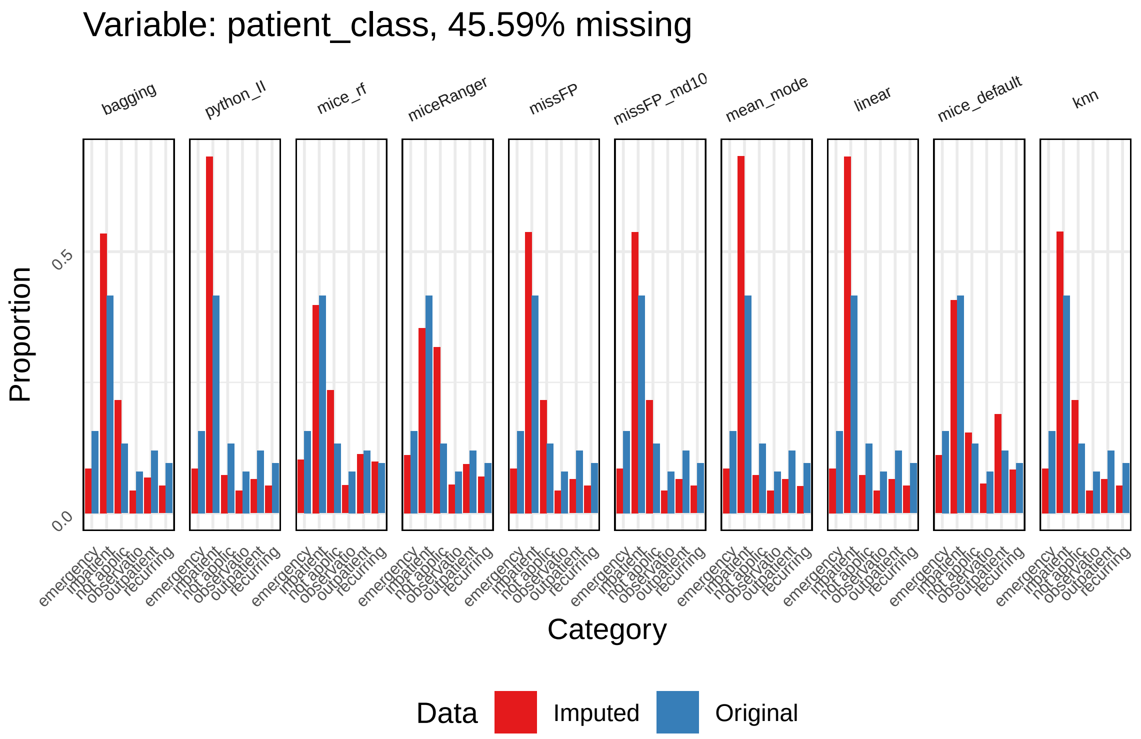

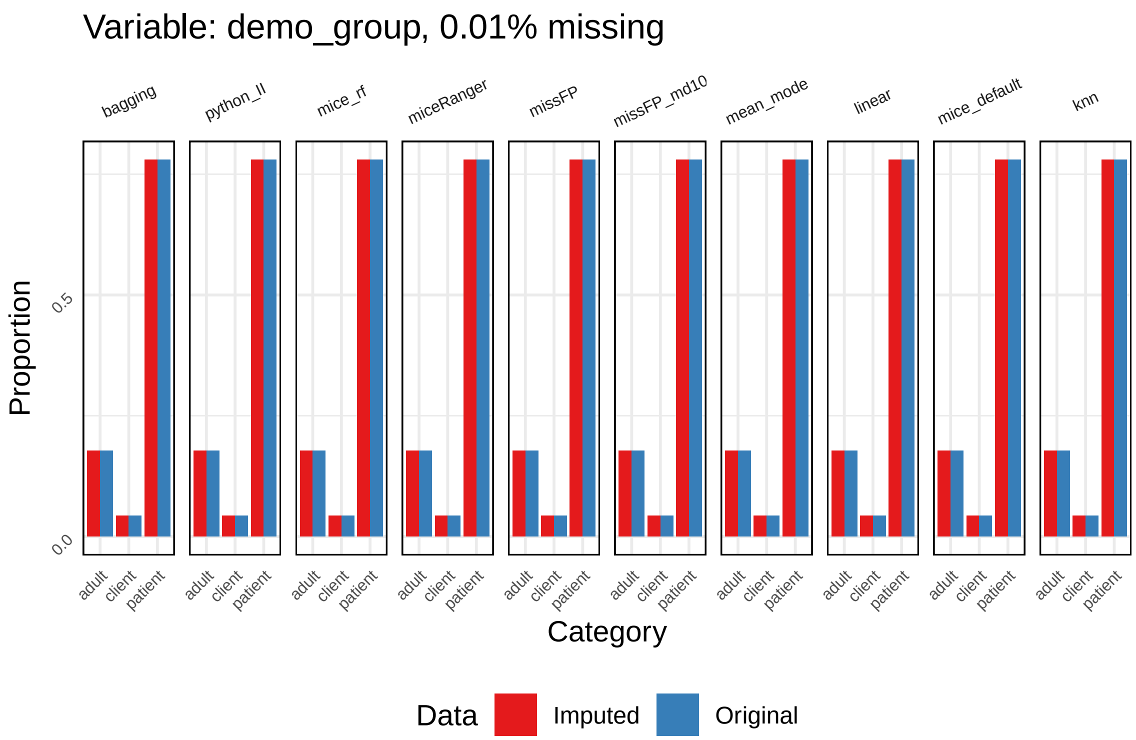

Variable: demo_group, 0.01% missing
bagging
python_ll
mice_rf
miceRanger
missFP
missFP_md10
mean_mode
linear
mice_default
knn
Proportion
0.0
0.5
adult
client
patient
Category
Data
Imputed
Original

CRASH-2 dataset

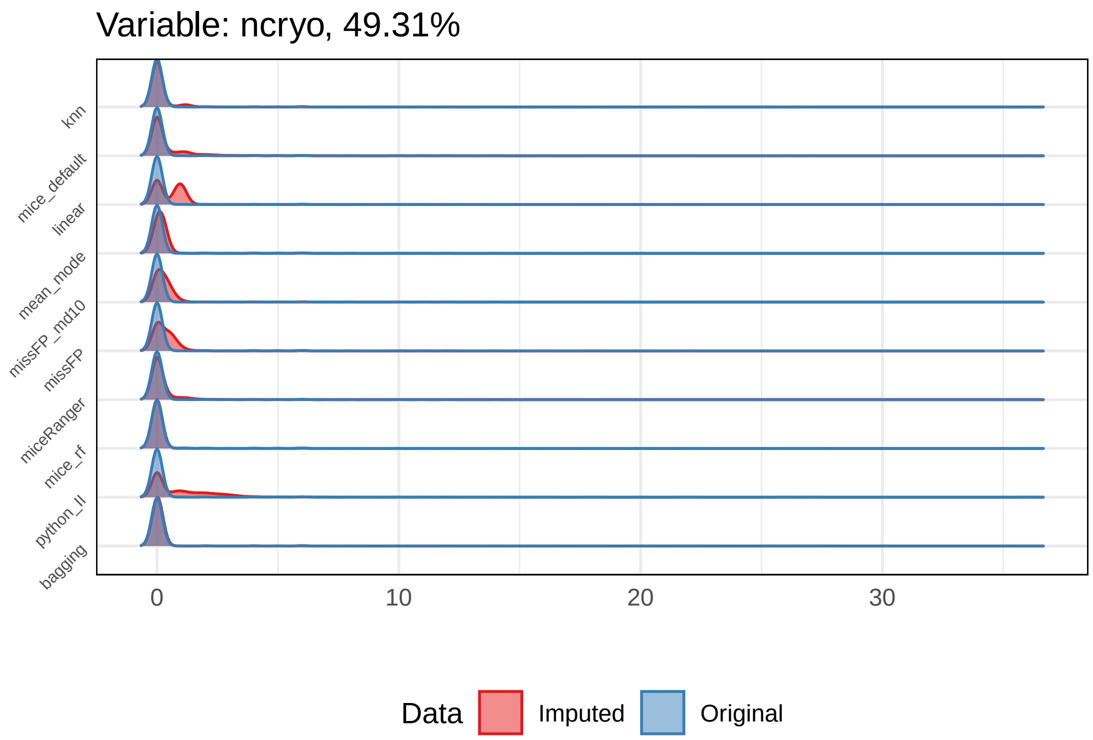

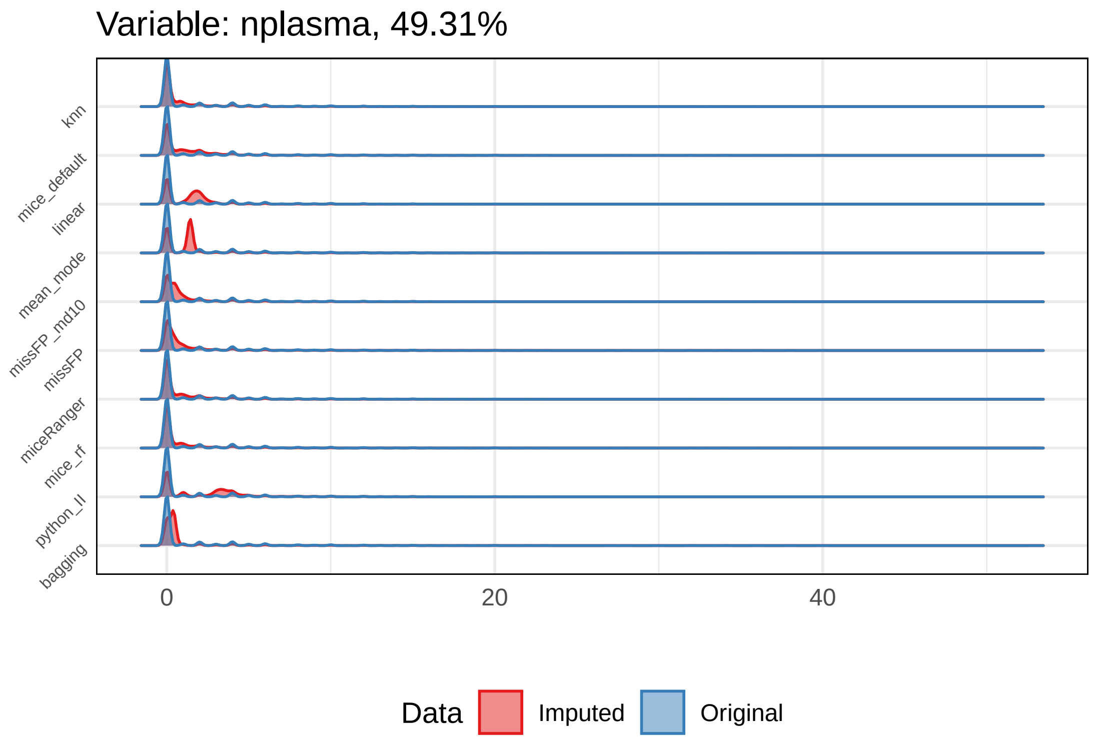

Variable: nplatelets, 49.31%

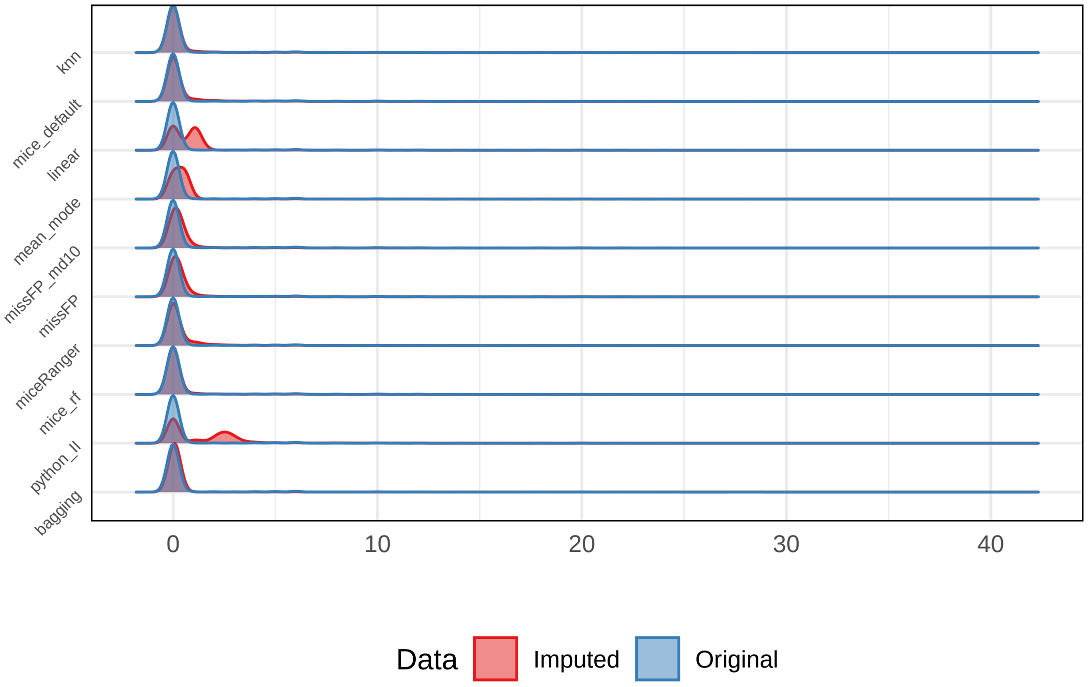

Variable: ncell, 49.3%

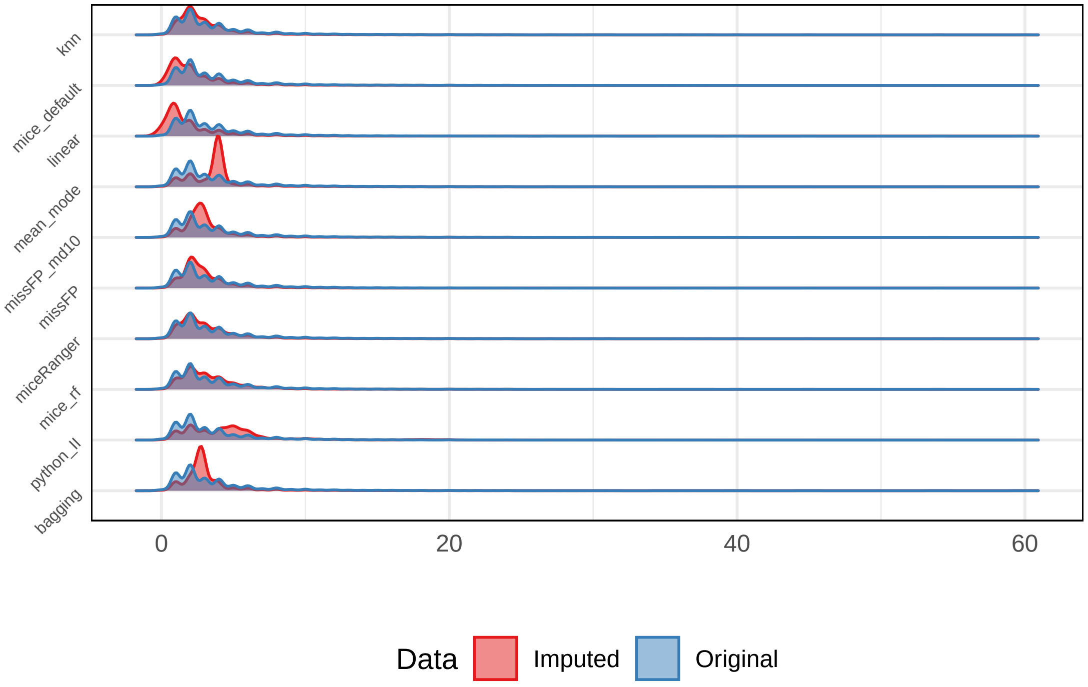

Variable: gcsverbal, 3.64%

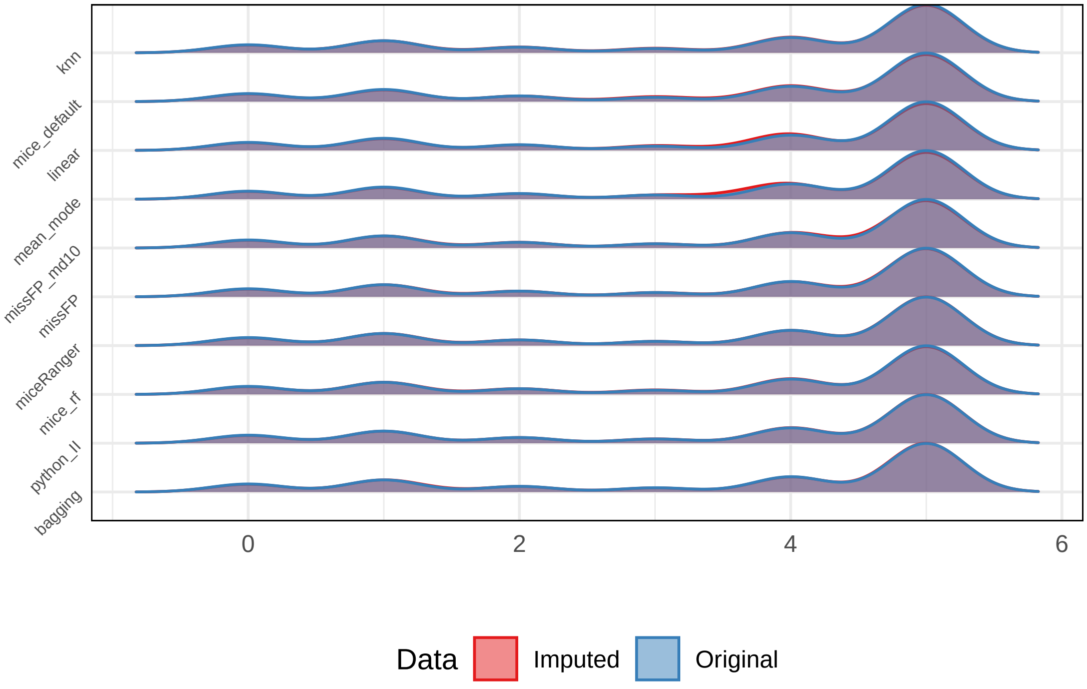

Variable: gcseye, 3.62%

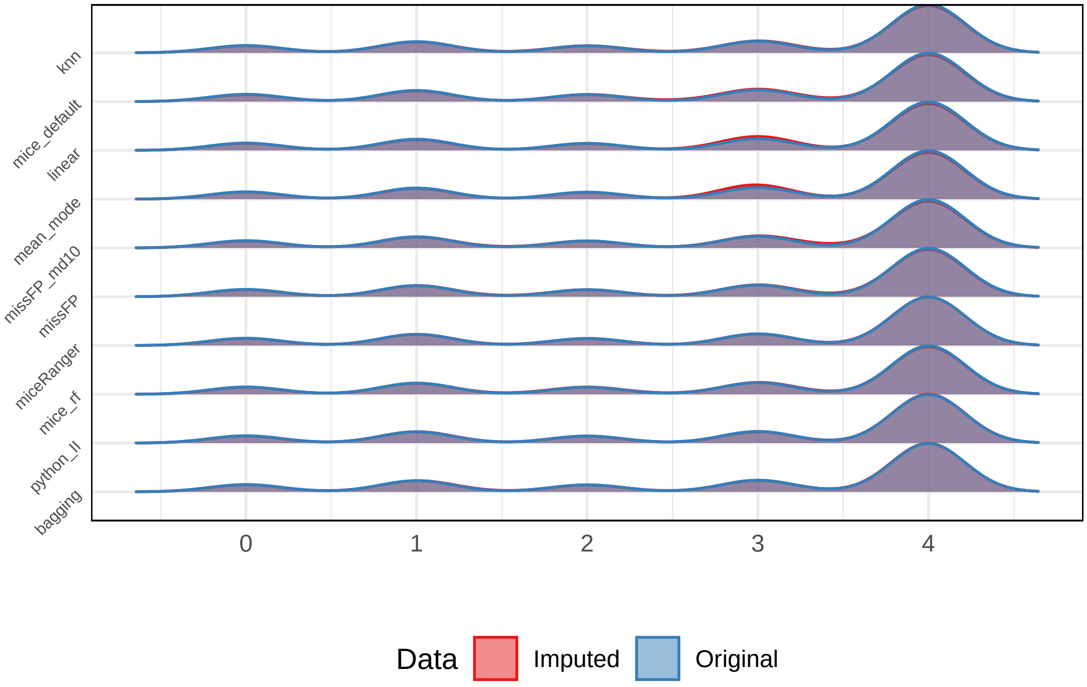

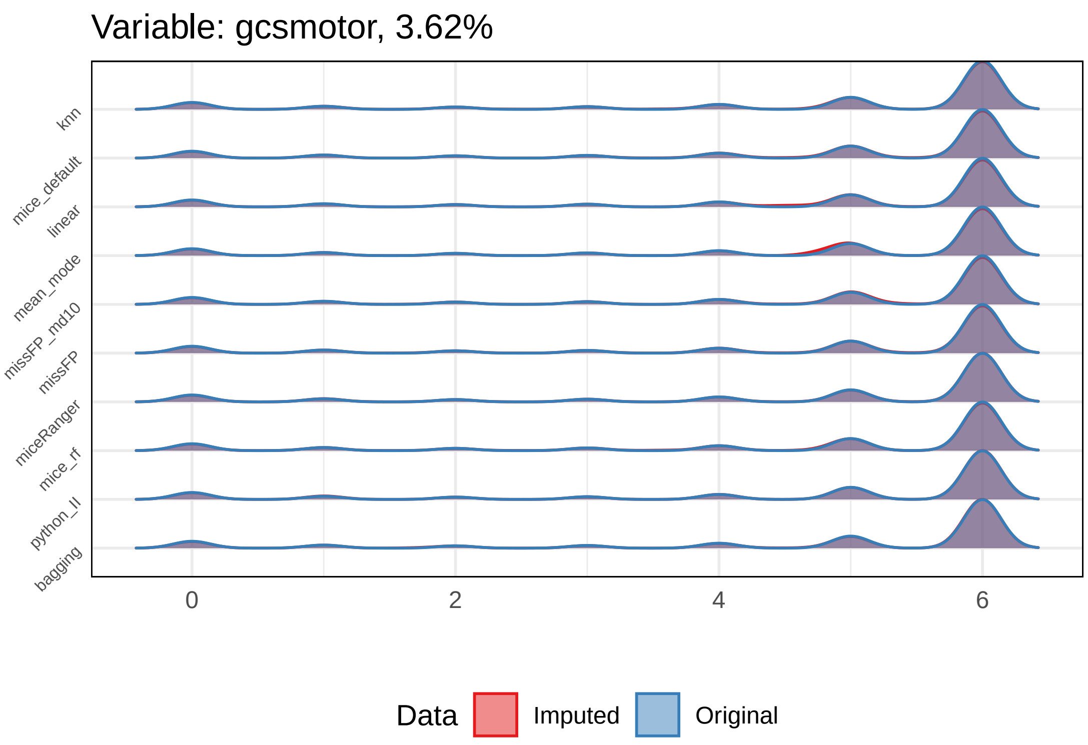
Variable: gcsmotor, 3.62%
knn
mice_default
linear
mean_mode
missFP _md10
missFP
miceRanger
mice_rf
python_II
bagging
0
2
4
6
Data
Imputed
Original

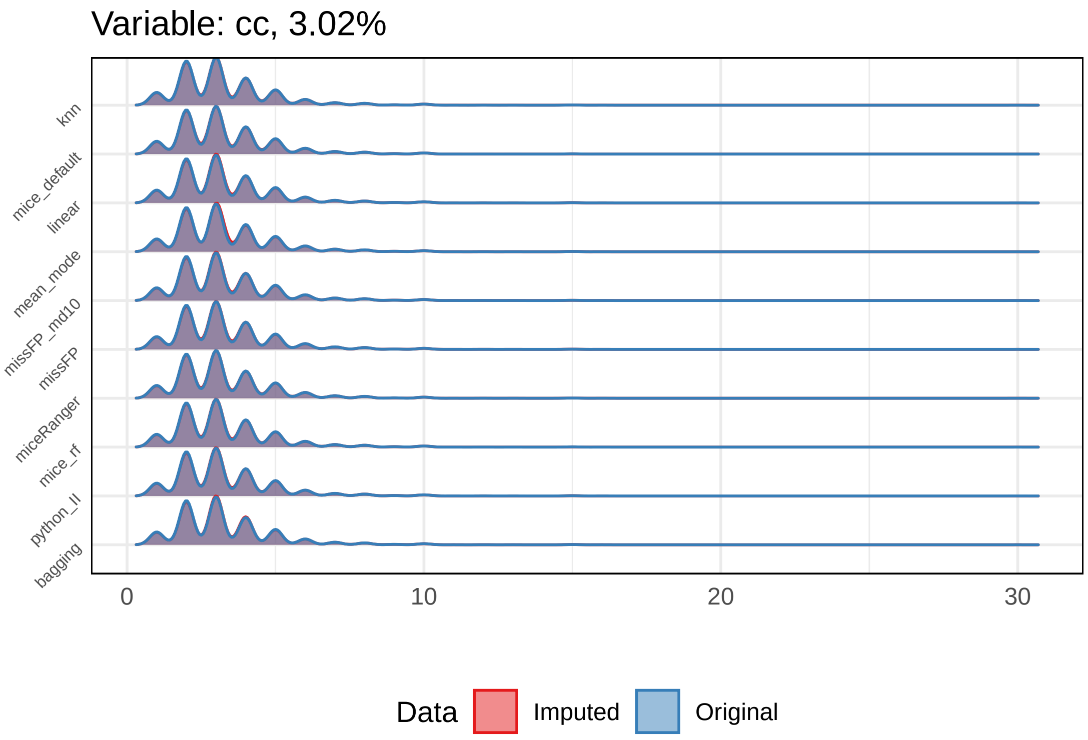
Variable: cc, 3.02%
knn
mice_default
linear
mean_mode
missFP _md10
missFP
miceRanger
mice_rf
python_II
bagging
0
10
20
30
Data
Imputed
Original

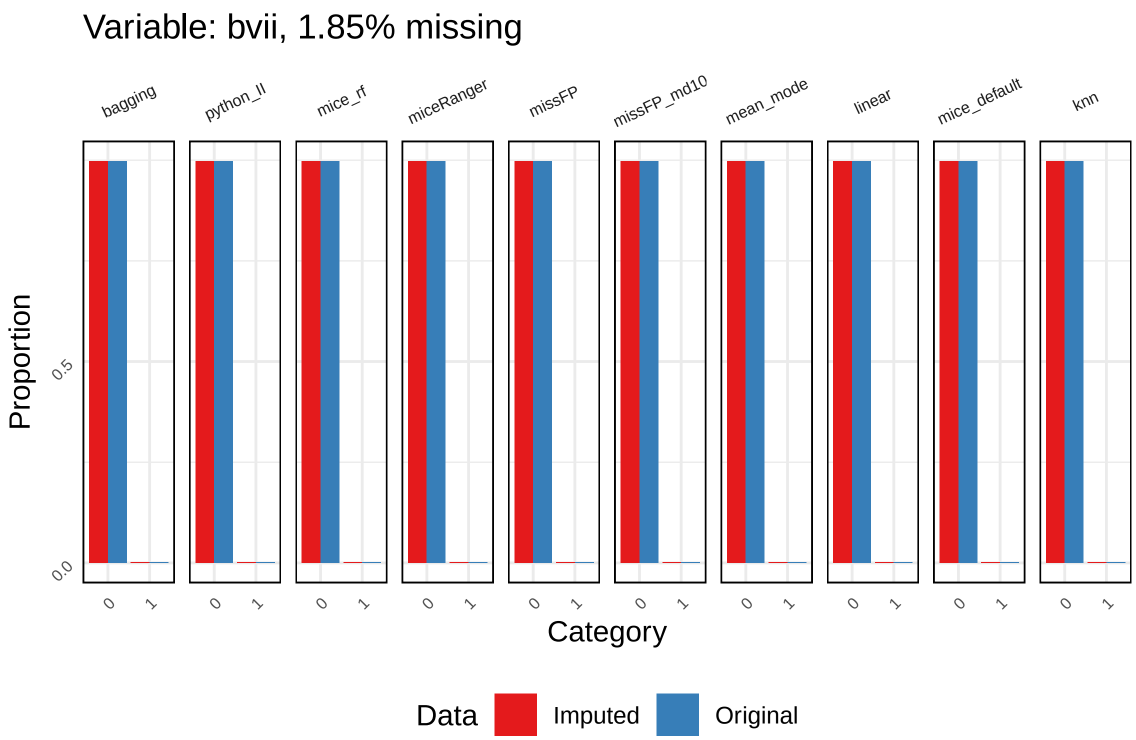
Variable: bvii, 1.85% missing
bagging
python_II
mice_rf
miceRanger
missFP
missFP_md10
mean_mode
linear
mice_default
knn
Proportion
0.0
0.5
0
1
Category
Data
Imputed
Original

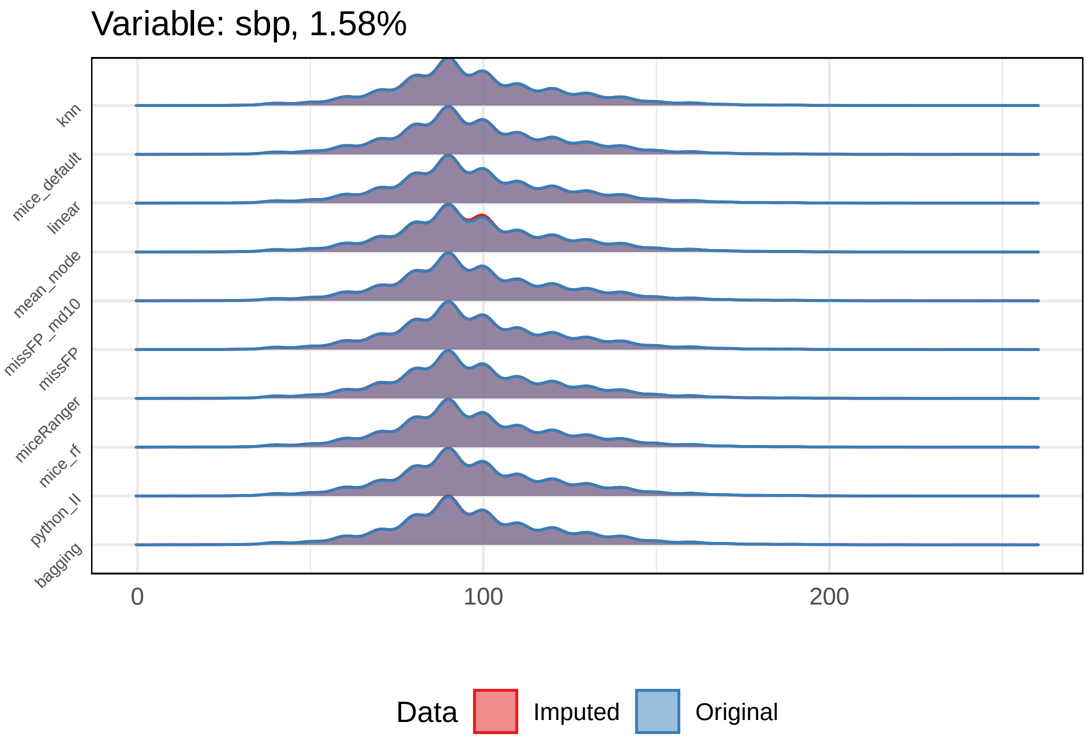
Variable: sbp, 1.58%
knn
mice_default
linear
mean_mode
missFP_md10
missFP
miceRanger
mice_rf
python_II
bagging
0
100
200
Data
Imputed
Original

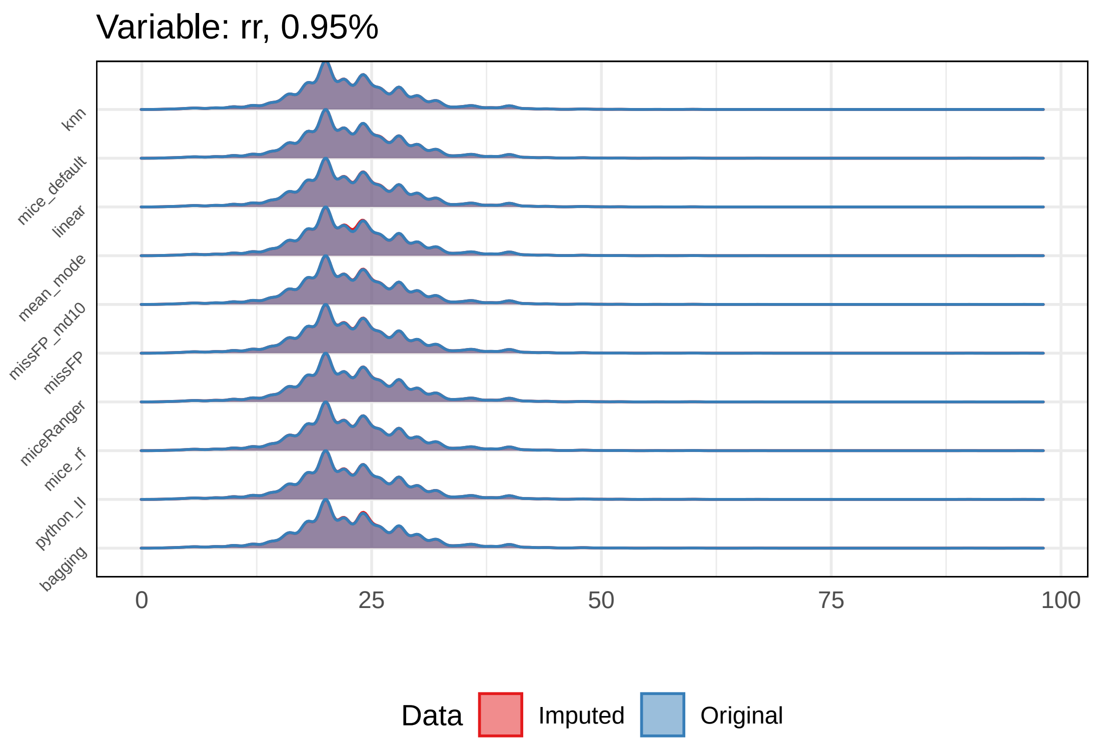
Variable: rr, 0.95%
knn
mice_default
linear
mean_mode
missFP_md10
missFP
miceRanger
mice_rf
python_II
bagging
0
25
50
75
100
Data
Imputed
Original

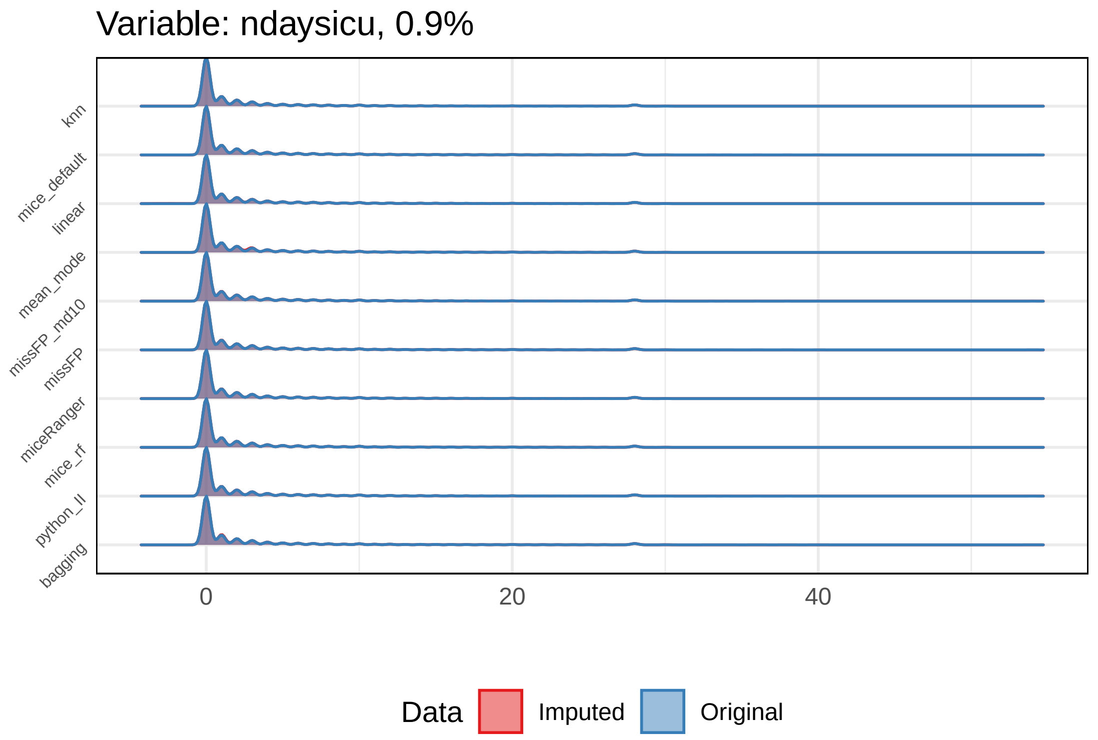
Variable: ndaysicu, 0.9%
knn
mice_default
linear
mean_mode
missFP_md10
missFP
miceRanger
mice_rf
python_II
bagging
0
20
40
Data
Imputed
Original

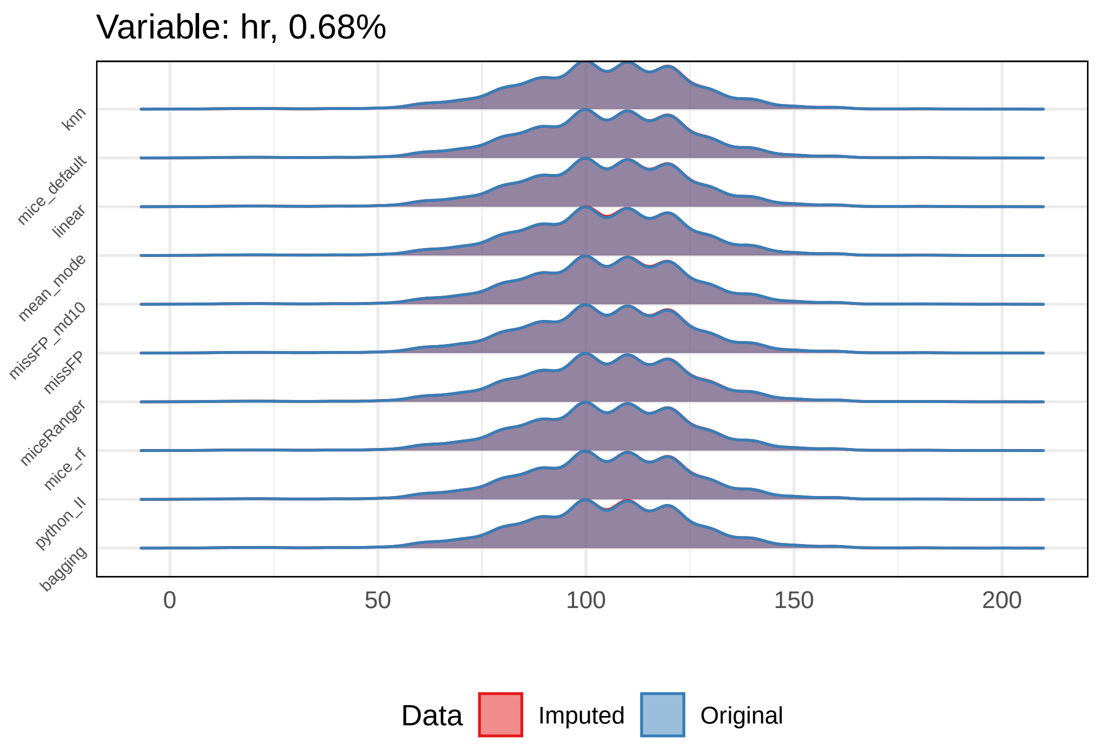

Variable: hr, 0.68%
knn
mice_default
linear
mean_mode
missFP_md10
missFP
miceRanger
mice_rf
python_II
bagging
0
50
100
150
200
Data
Imputed
Original

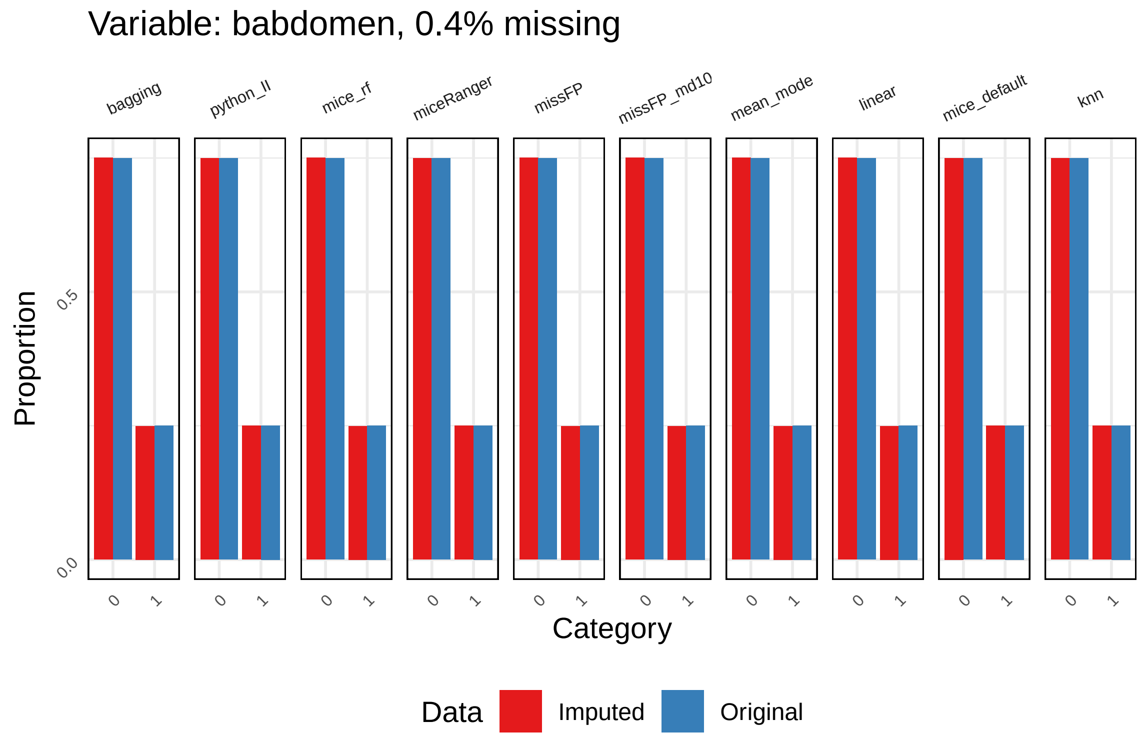

Variable: babdomen, 0.4% missing
bagging
python_II
mice_rf
miceRanger
missFP
missFP_md10
mean_mode
linear
mice_default
knn
Proportion
0.5
0.0
0
1
Category
Data
Imputed
Original

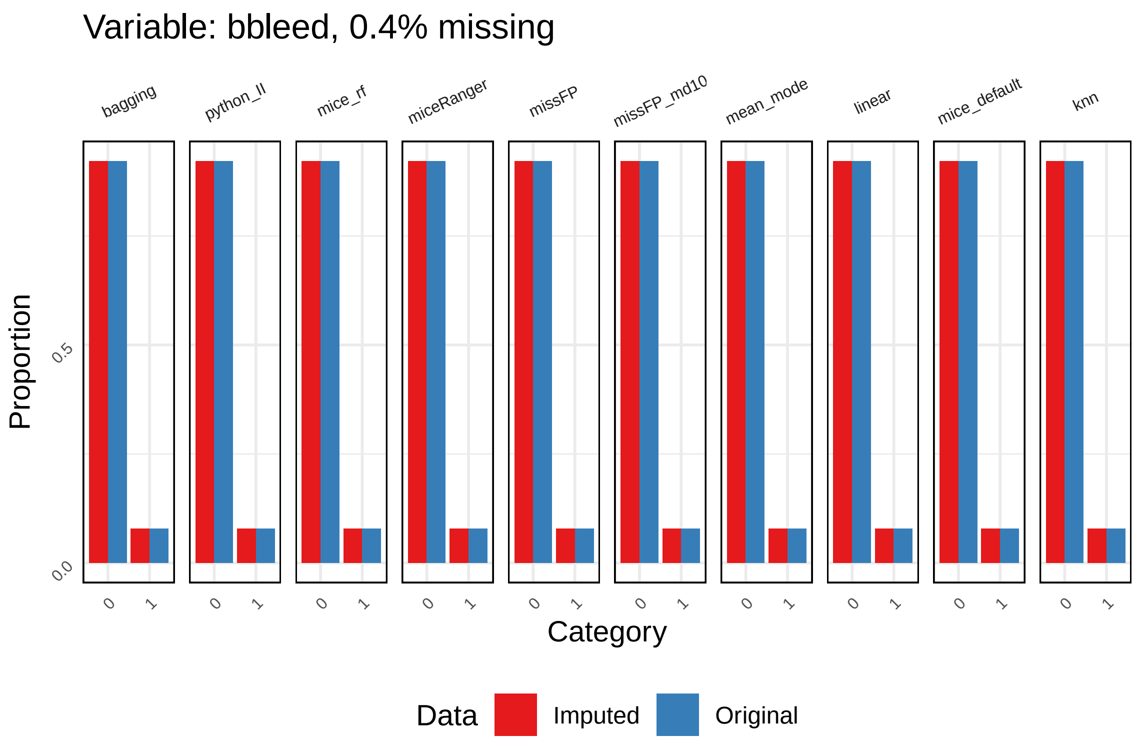
Variable: bbleed, 0.4% missing
bagging
python_II
mice_rf
miceRanger
missFP
missFP_md10
mean_mode
linear
mice_default
knn
Proportion
0.0
0.5
0
1
Category
Data
Imputed
Original

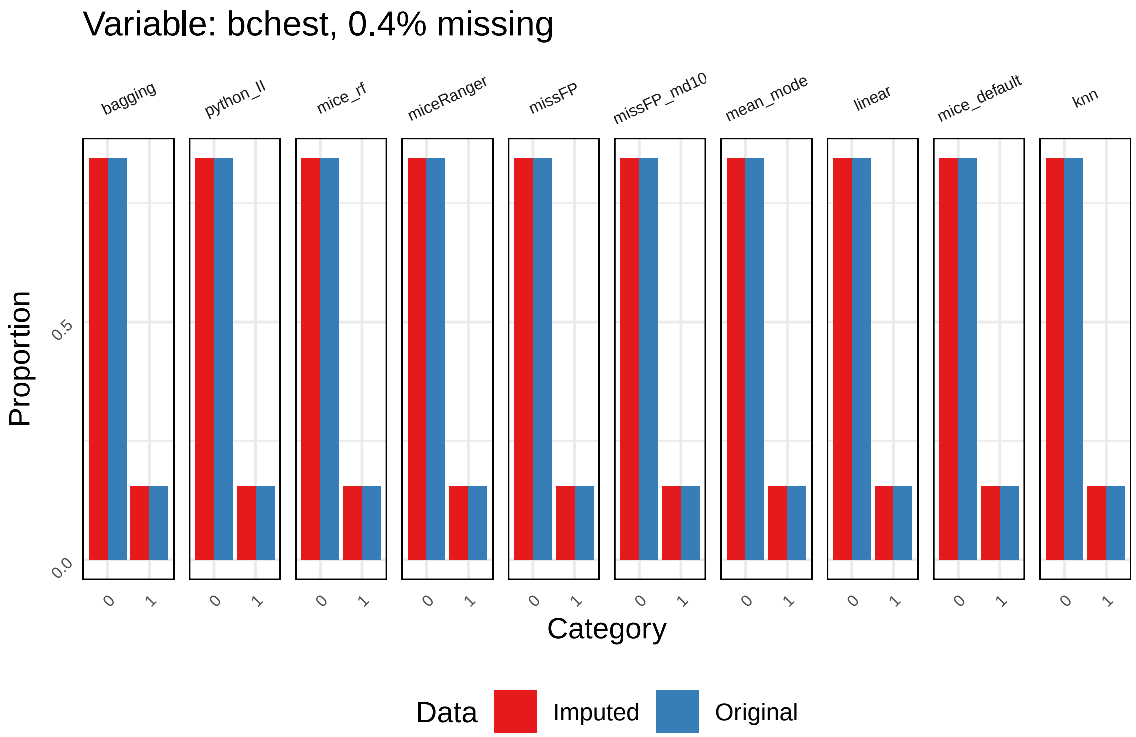
Variable: bchest, 0.4% missing
bagging
python_II
mice_rf
miceRanger
missFP
missFP_md10
mean_mode
linear
mice_default
knn
Proportion
0.0
0.5
0
1
Category
Data
Imputed
Original

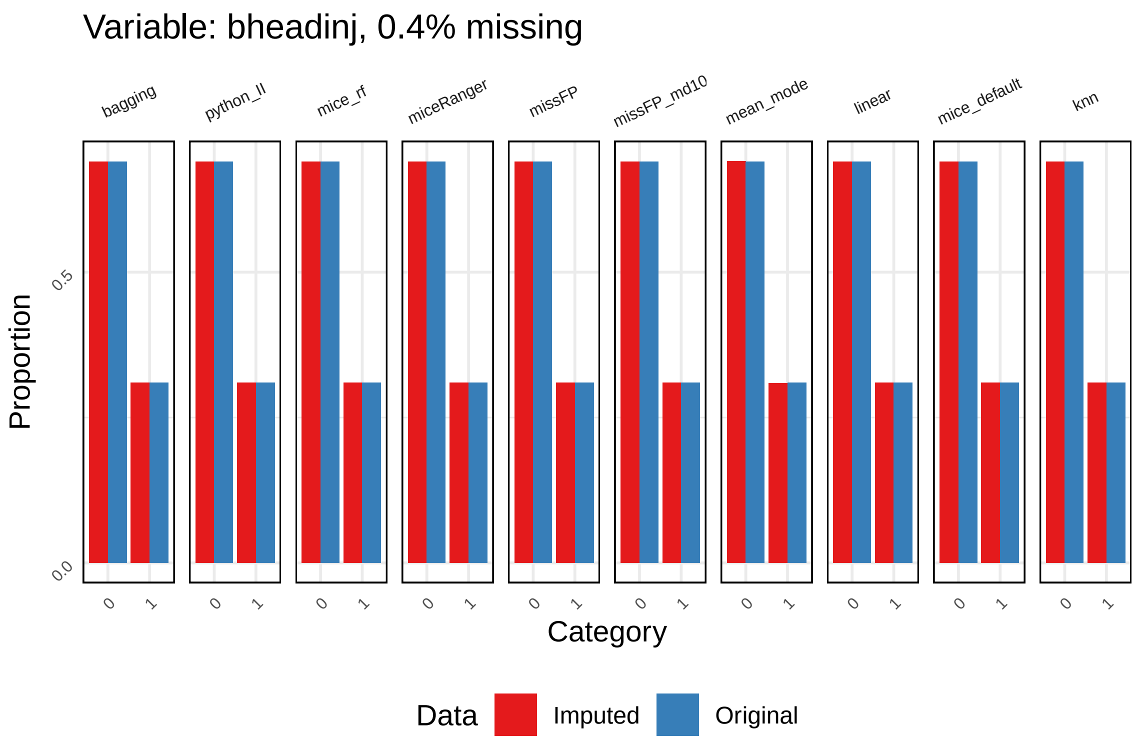
Variable: bheadinj, 0.4% missing
bagging
python_ll
mice_rf
miceRanger
missFP
missFP_md10
mean_mode
linear
mice_default
knn
Proportion
0.0
0.5
0
1
Category
Data
Imputed
Original

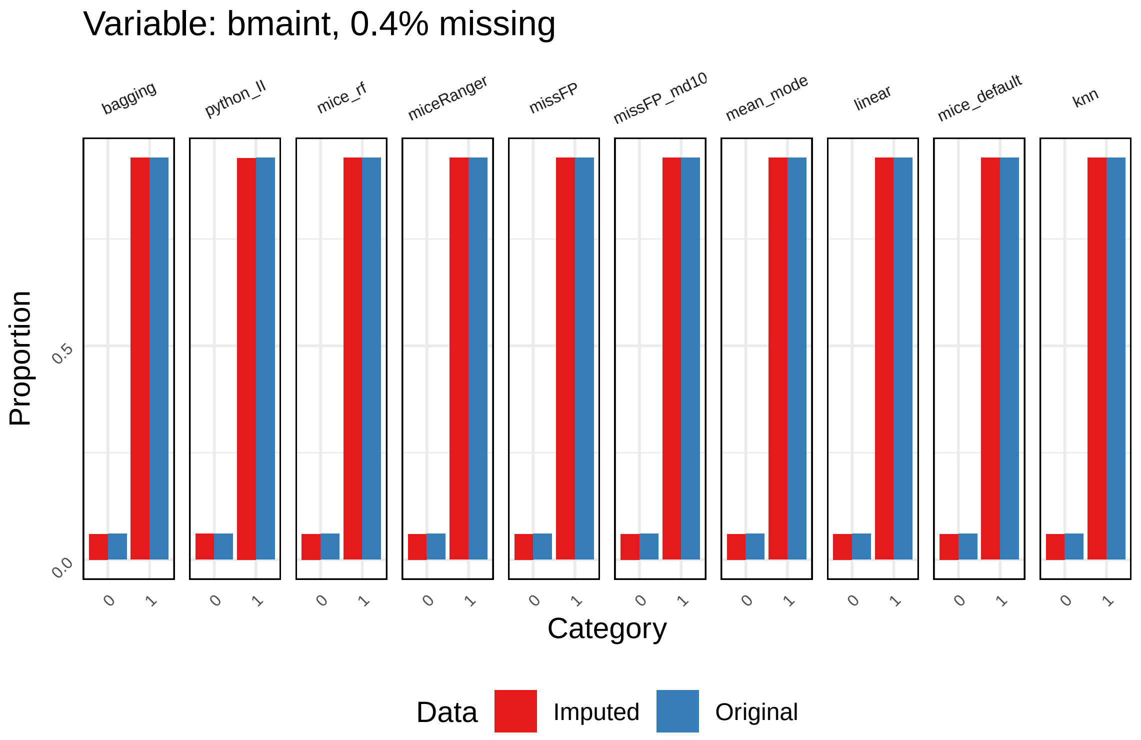
Variable: bmaint, 0.4% missing
bagging
python_ll
mice_rf
miceRanger
missFP
missFP_md10
mean_mode
linear
mice_default
knn
Proportion
0.0
0.5
0
1
Category
Data
Imputed
Original

Variable: bneuro, 0.4% missing

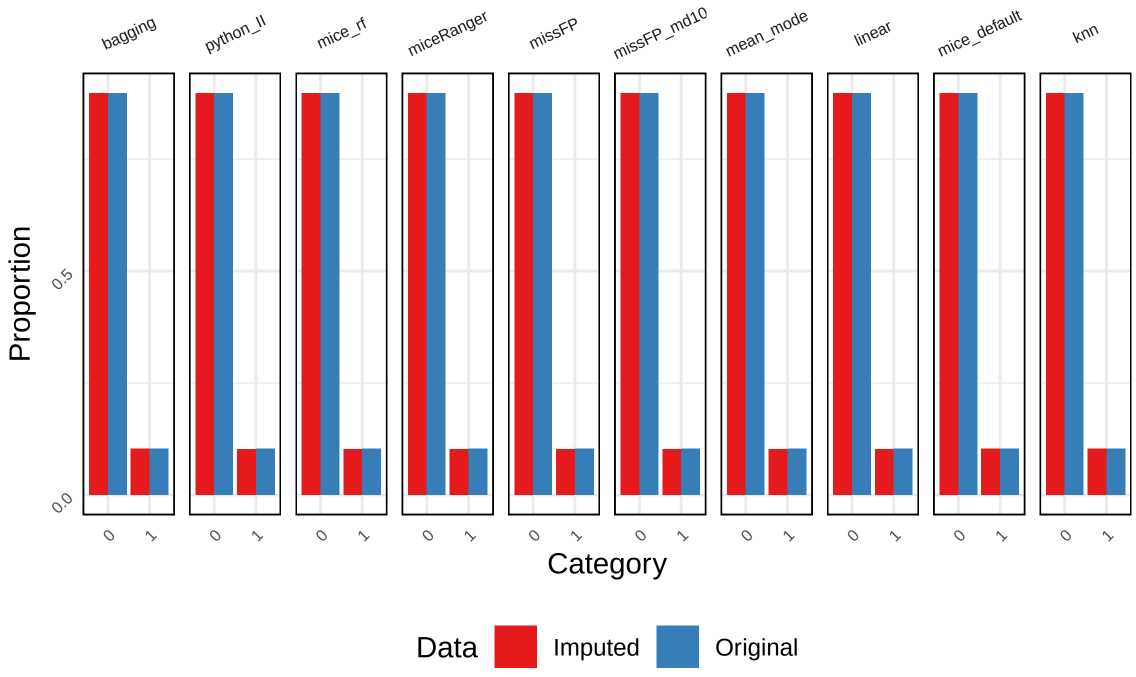

Variable: bpelvis, 0.4% missing

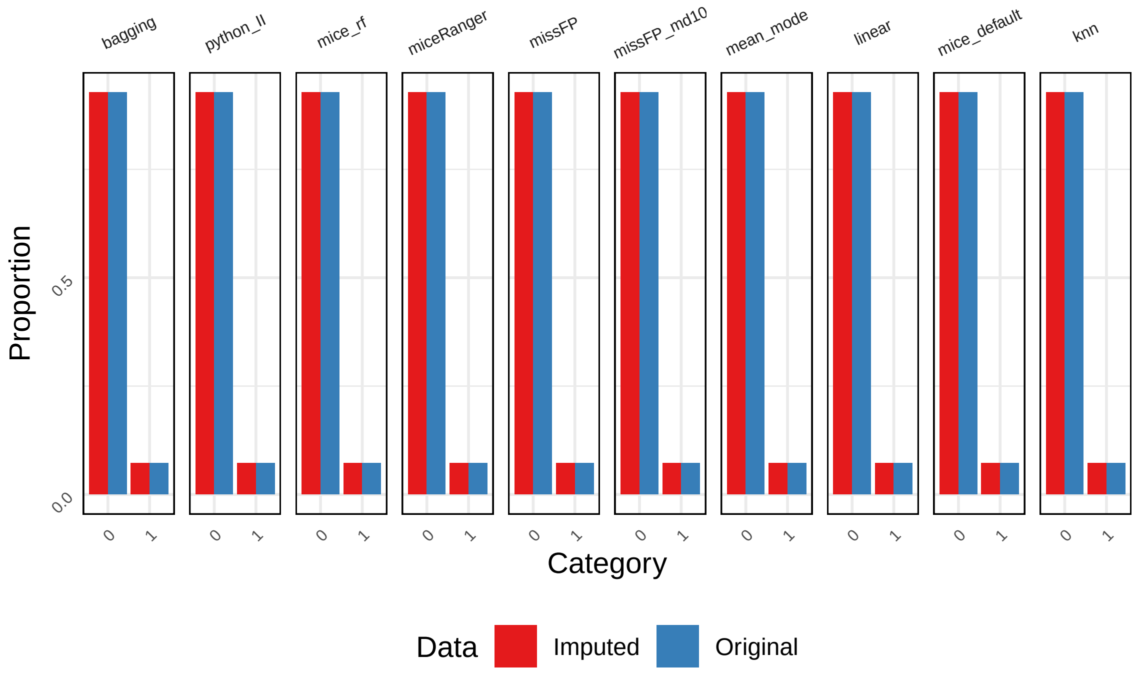

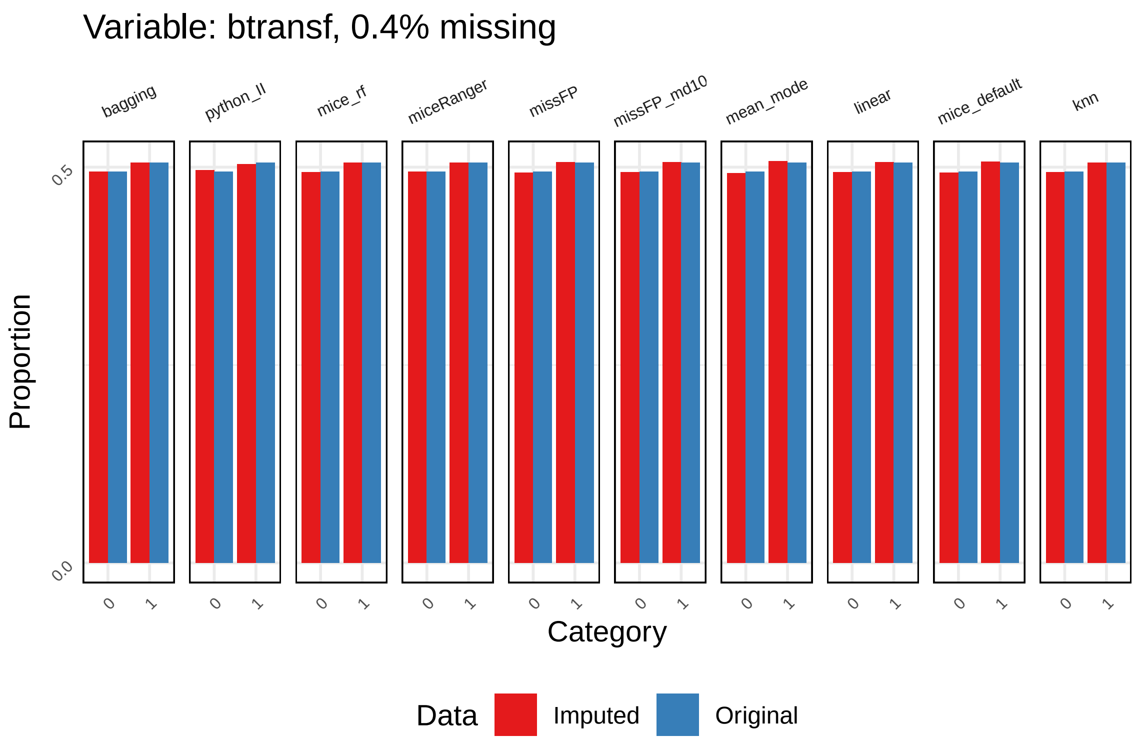
Variable: btransf, 0.4% missing
bagging
python_II
mice_rf
miceRanger
missFP
missFP_md10
mean_mode
linear
mice_default
knn
Proportion
0.5
0.0
0
1
Category
Data
Imputed
Original

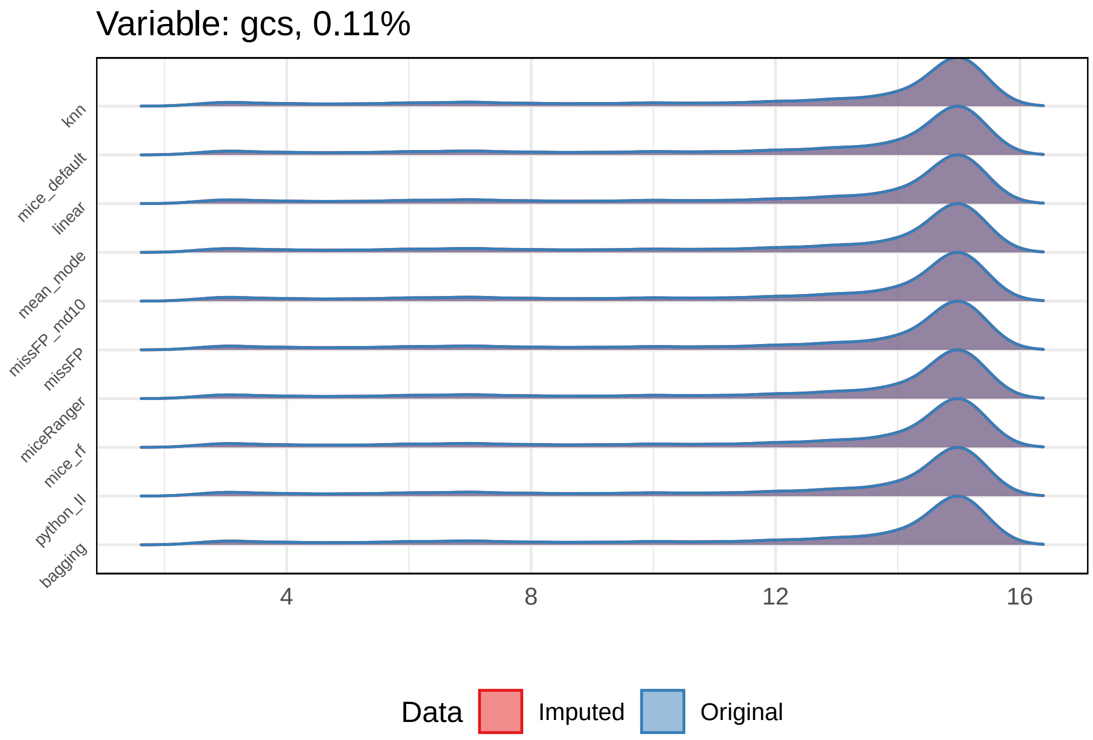
Variable: gcs, 0.11%
knn
mice_default
linear
mean_mode
missFP_md10
missFP
miceRanger
mice_rf
python_II
bagging
4
8
12
16
Data
Imputed
Original

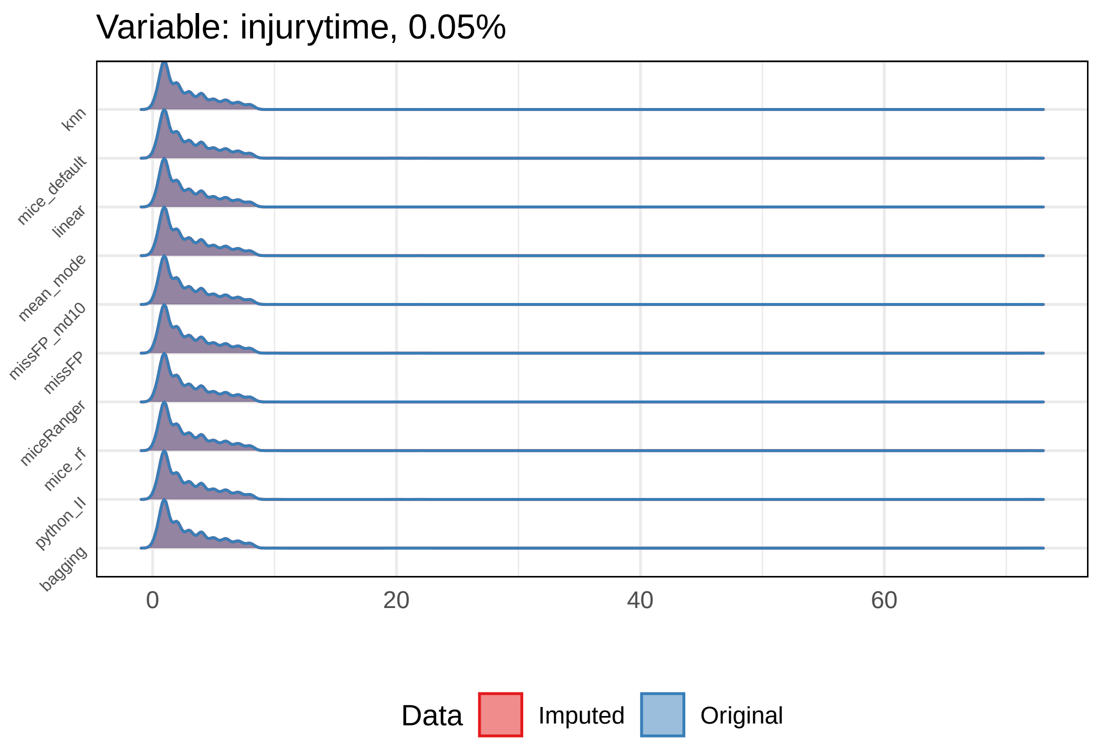
Variable: injurytime, 0.05%
knn
mice_default
linear
mean_mode
missFP_md10
missFP
miceRanger
mice_rf
python_II
bagging
0
20
40
60
Data
Imputed
Original

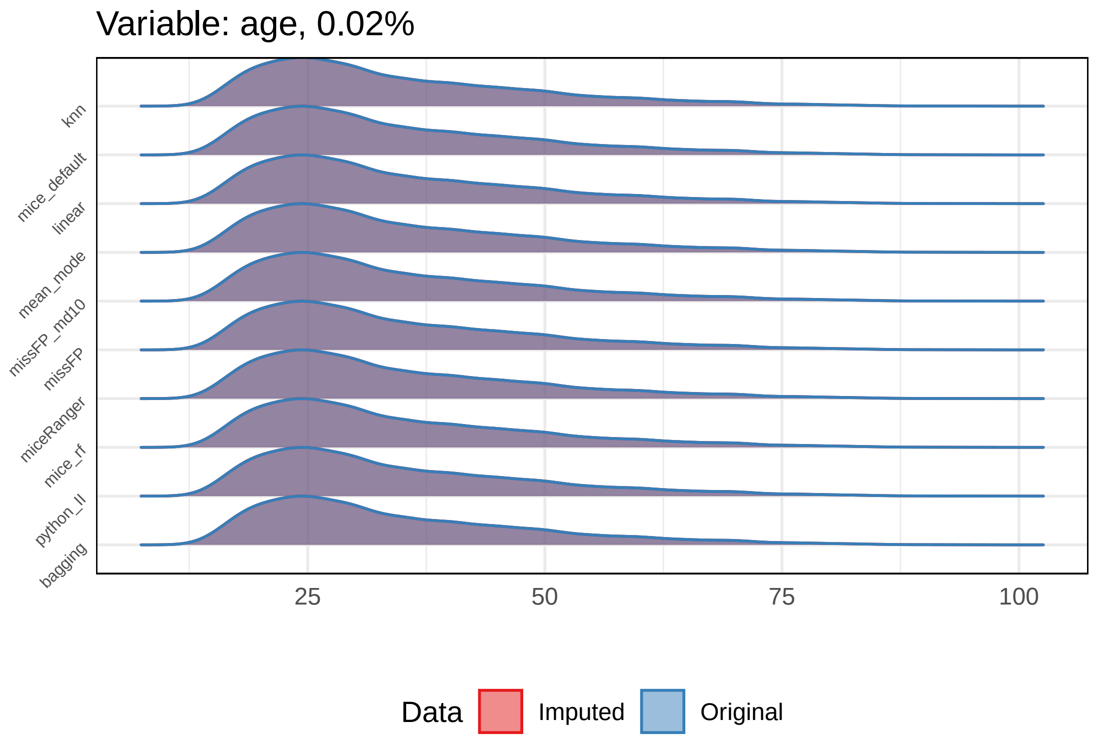
Variable: age, 0.02%
knn
mice_default
linear
mean_mode
missFP_md10
missFP
miceRanger
mice_rf
python_II
bagging
25
50
75
100
Data
Imputed
Original

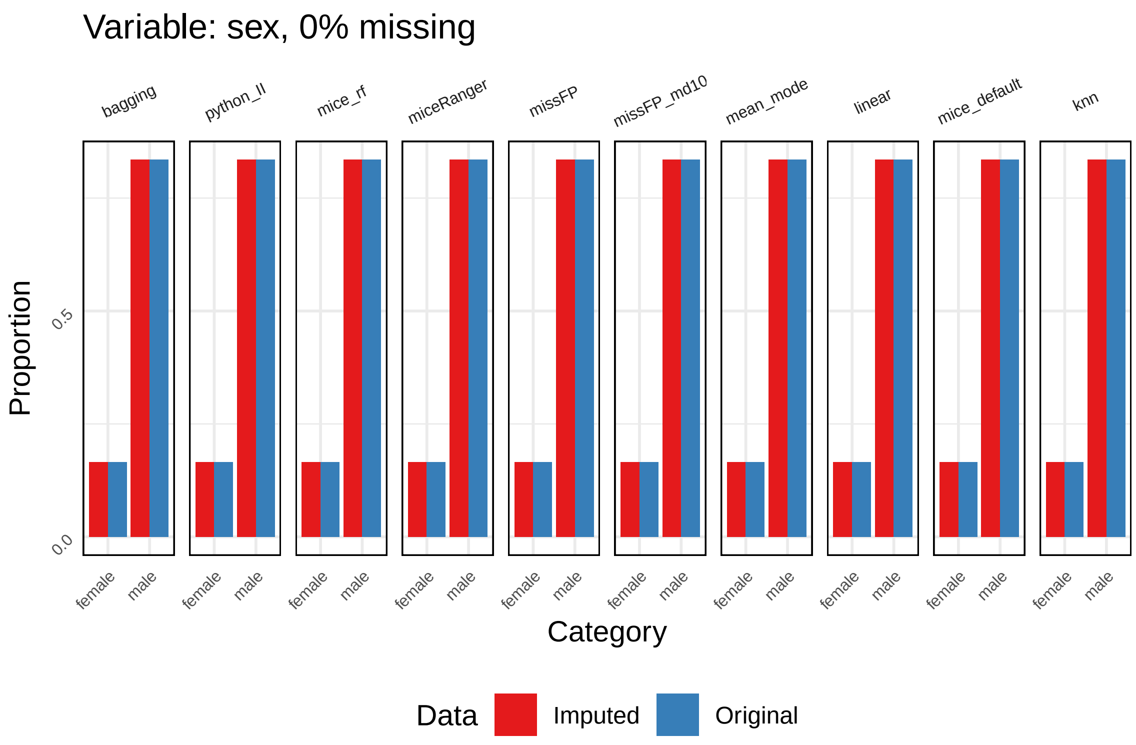
Variable: sex, 0% missing
bagging
python_ll
mice_rf
miceRanger
missFP
missFP_md10
mean_mode
linear
mice_default
knn
Proportion
0.0
0.5
female
male
Category
Data
Imputed
Original

IST dataset

Variable: RDEF5, 20.29% missing

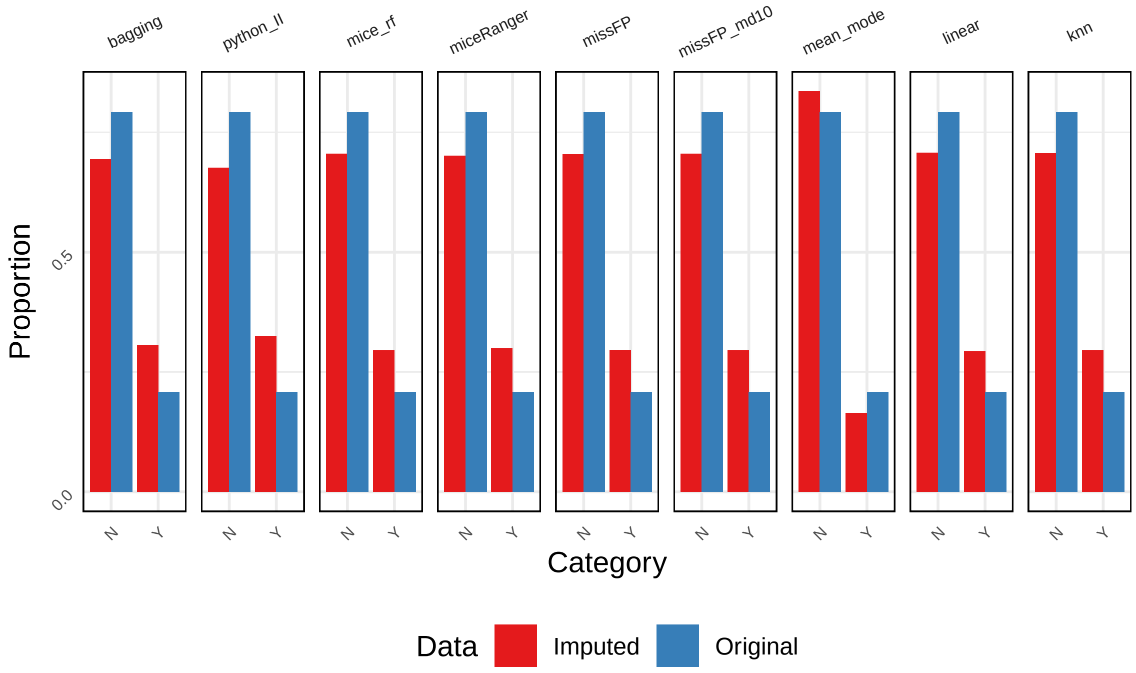

Variable: RDEF6, 17.75% missing

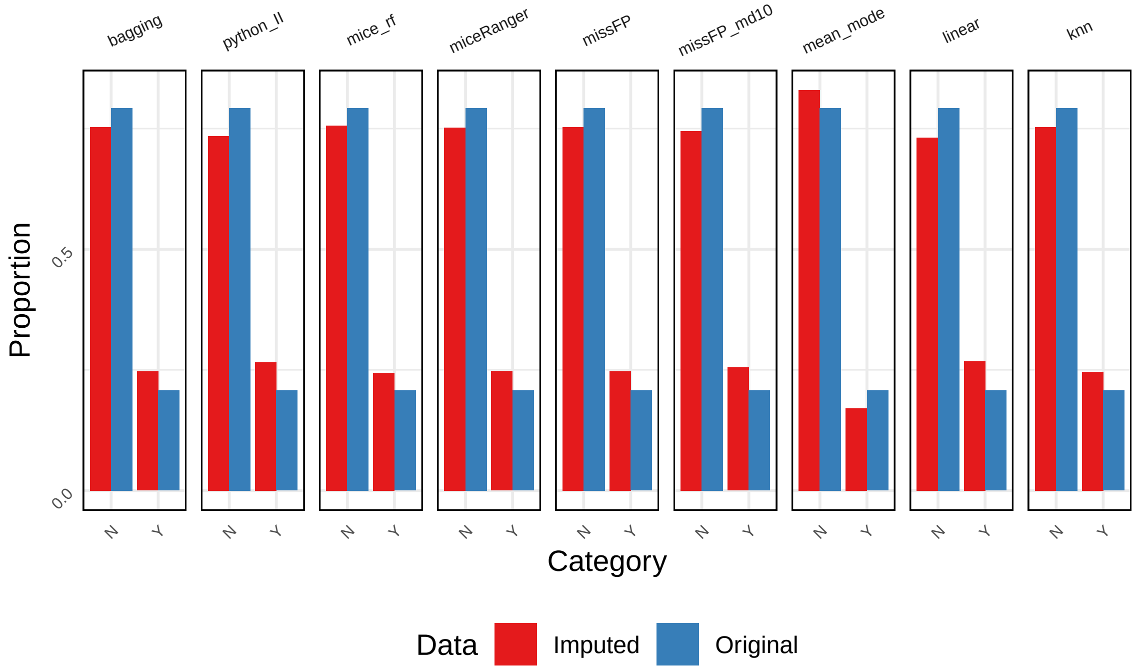

Variable: RDEF7, 8.19% missing

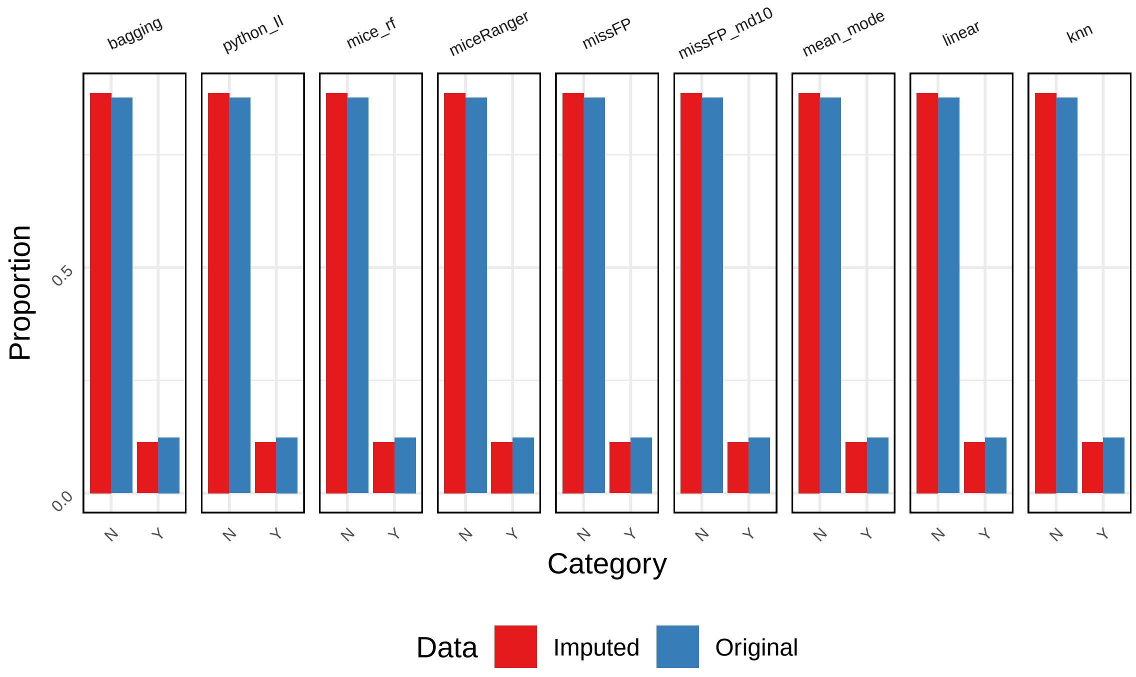

Variable: RDEF8, 6.43% missing

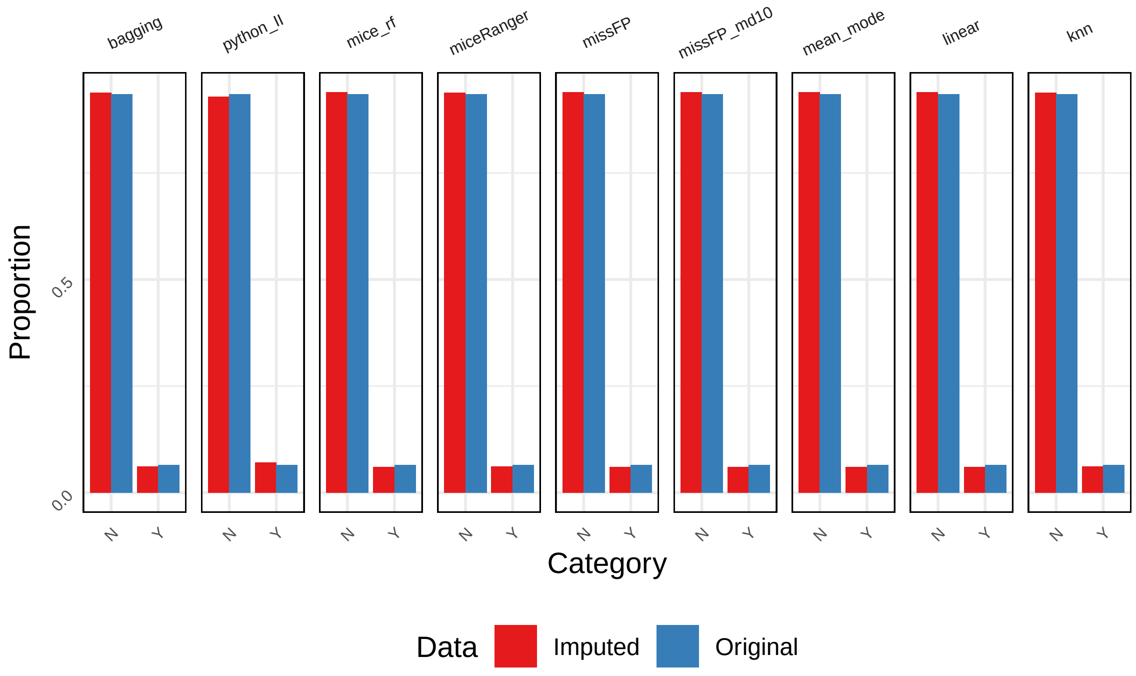

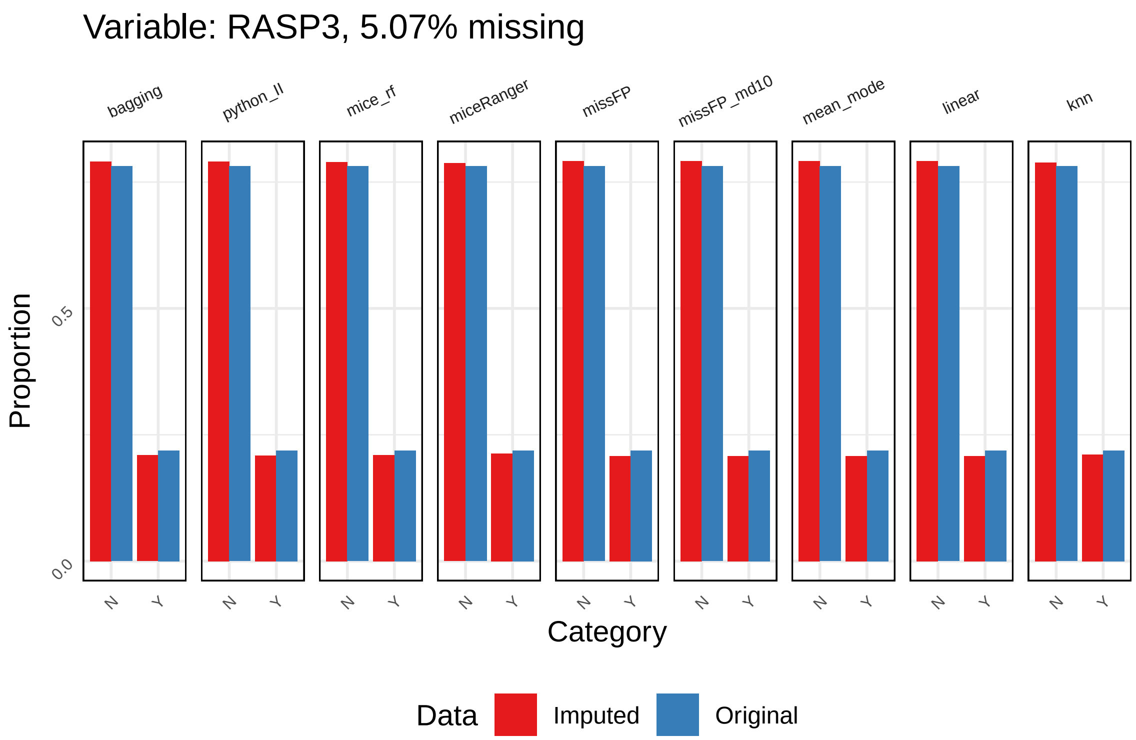
Variable: RASP3, 5.07% missing
bagging
python_II
mice_rf
miceRanger
missFP
missFP_md10
mean_mode
linear
knn
Proportion
0.0
0.5
N
Y
Category
Data
Imputed
Original

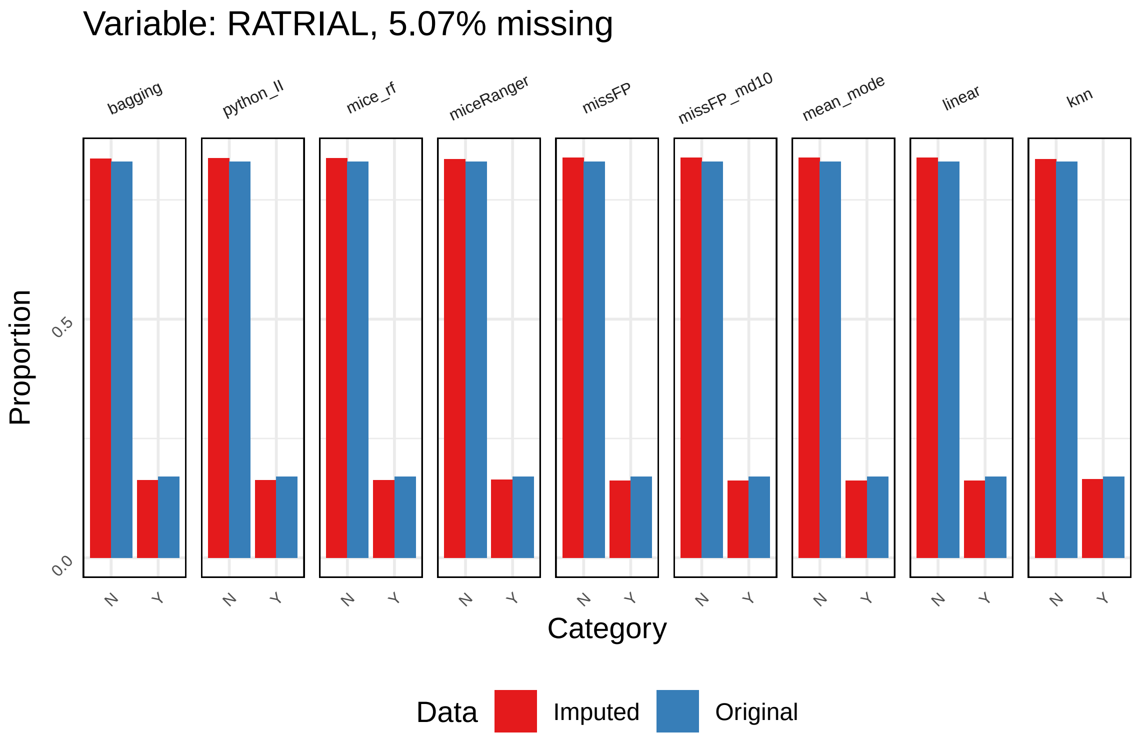
Variable: RATRIAL, 5.07% missing
bagging
python_II
mice_rf
miceRanger
missFP
missFP_md10
mean_mode
linear
knn
Proportion
0.0
0.5
N
Y
Category
Data
Imputed
Original

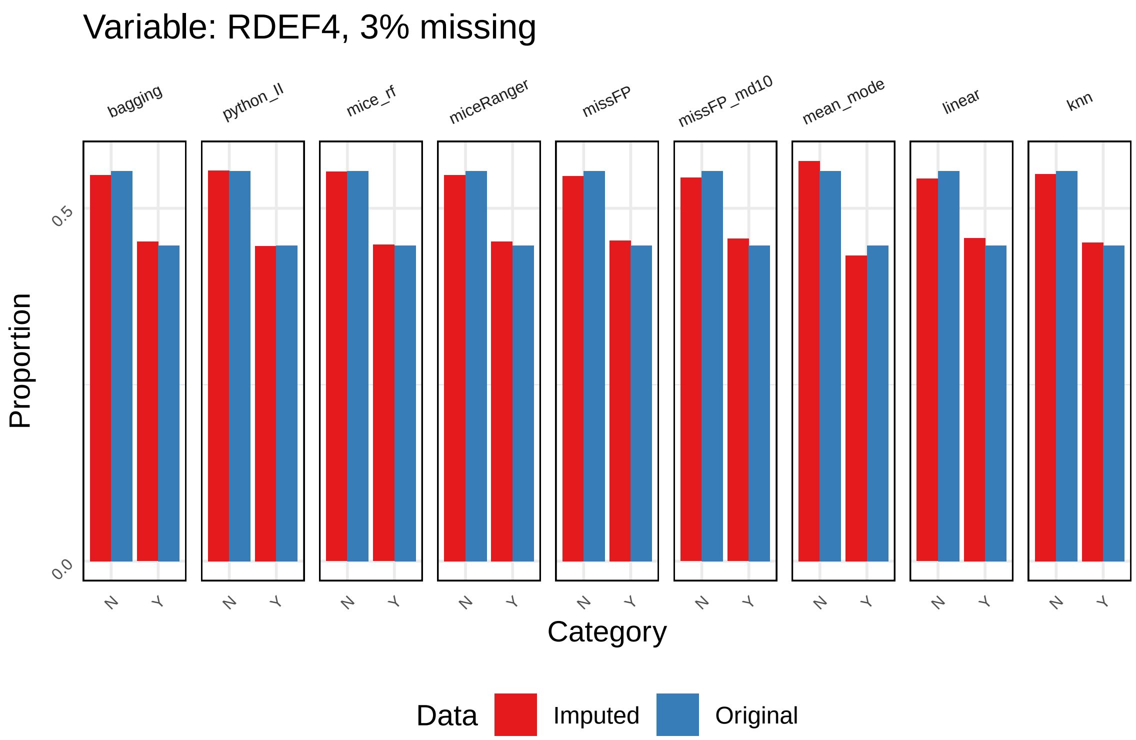
Variable: RDEF4, 3% missing
bagging
python_II
mice_rf
miceRanger
missFP
missFP_md10
mean_mode
linear
knn
Proportion
0.5
0.0
N
Y
Category
Data
Imputed
Original

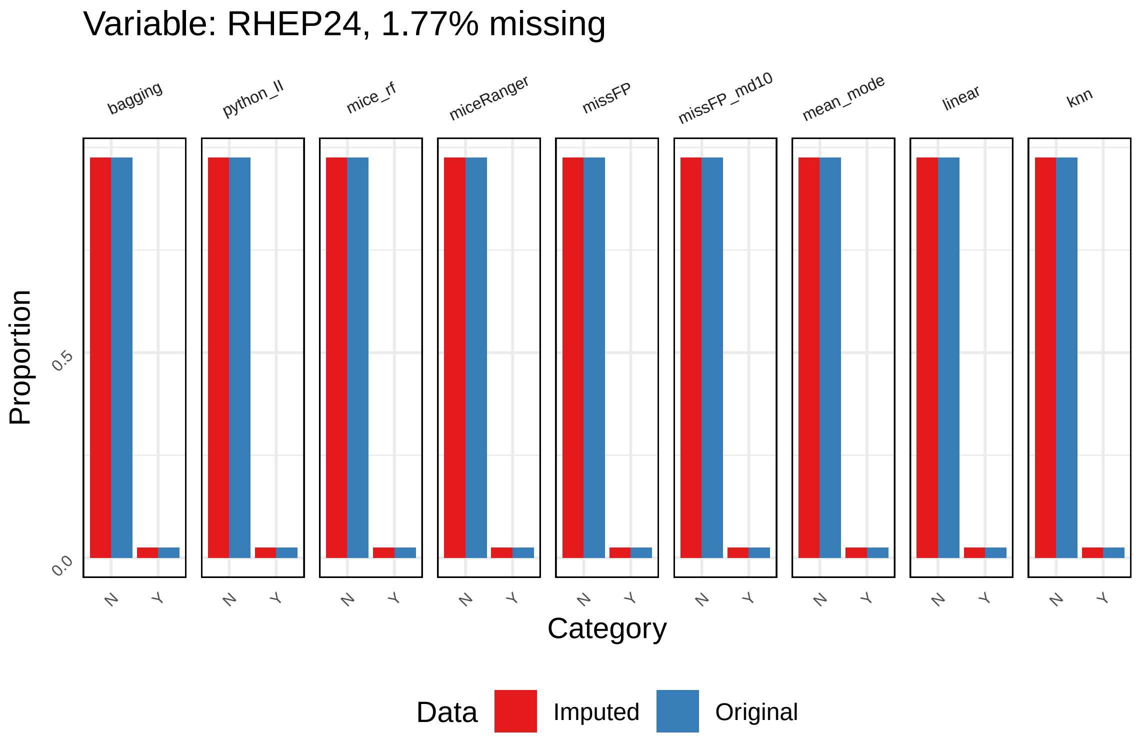
Variable: RHEP24, 1.77% missing
bagging
python_II
mice_rf
miceRanger
missFP
missFP_md10
mean_mode
linear
knn
Proportion
0.5
0.0
N
Y
Category
Data
Imputed
Original

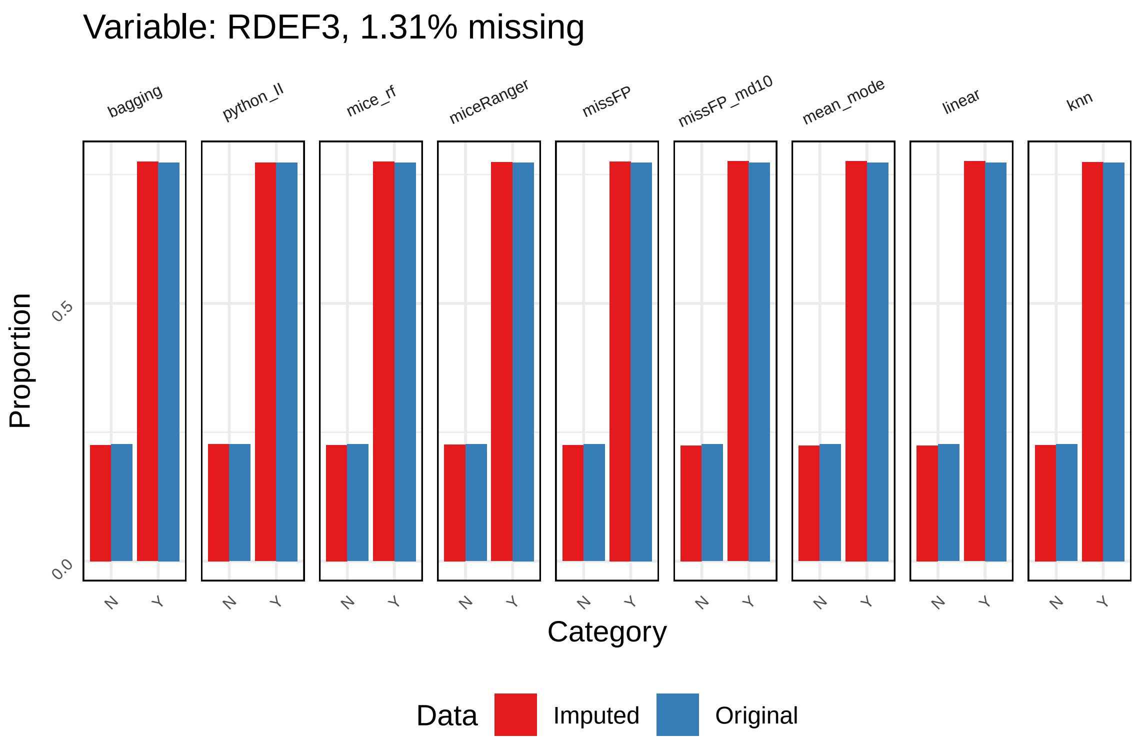
Variable: RDEF3, 1.31% missing
bagging
python_II
mice_rf
miceRanger
missFP
missFP_md10
mean_mode
linear
knn
Proportion
0.5
0.0
N
Y
Category
Data
Imputed
Original

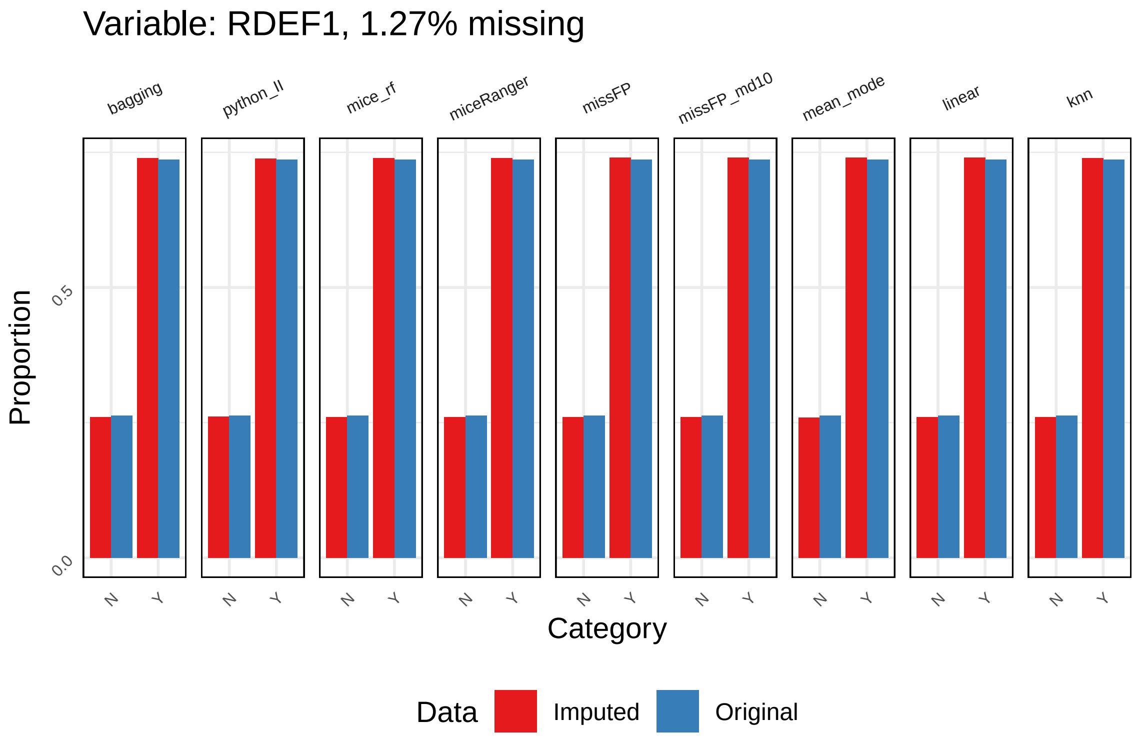
Variable: RDEF1, 1.27% missing
bagging
python_II
mice_rf
miceRanger
missFP
missFP_md10
mean_mode
linear
knn
Proportion
0.5
0.0
N
Y
Category
Data
Imputed
Original

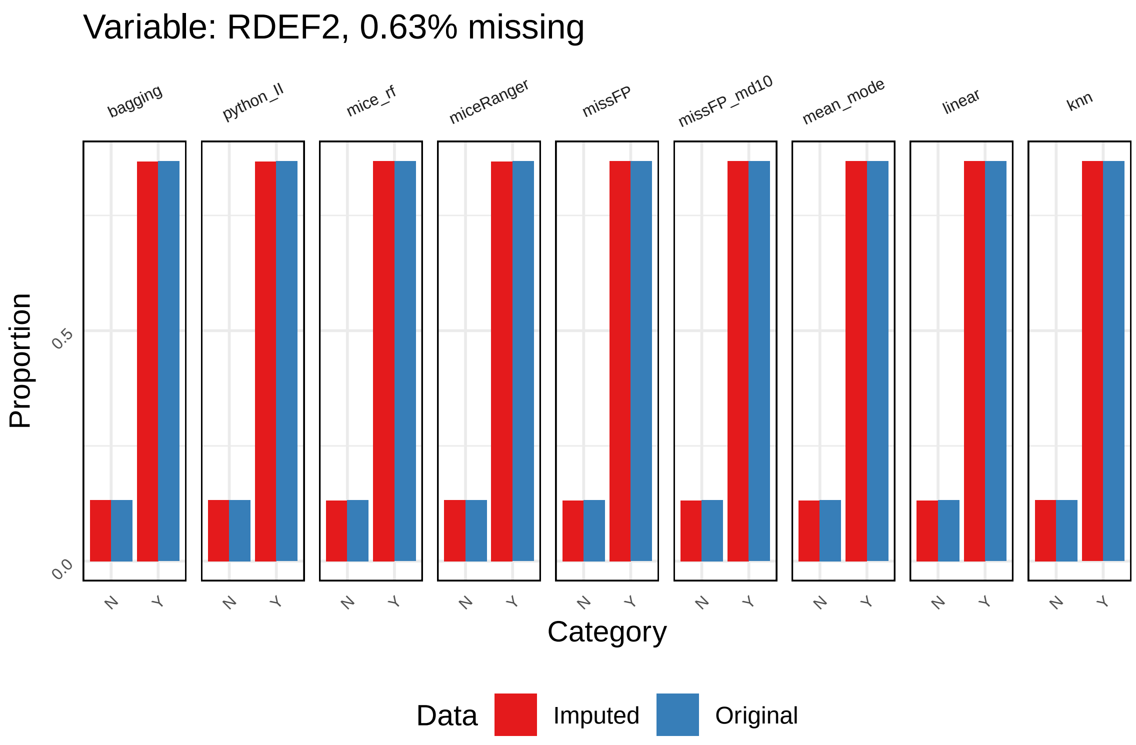
Variable: RDEF2, 0.63% missing
bagging
python_II
mice_rf
miceRanger
missFP
missFP_md10
mean_mode
linear
knn
Proportion
0.5
0.0
N
Y
Category
Data
Imputed
Original

Diabetes dataset

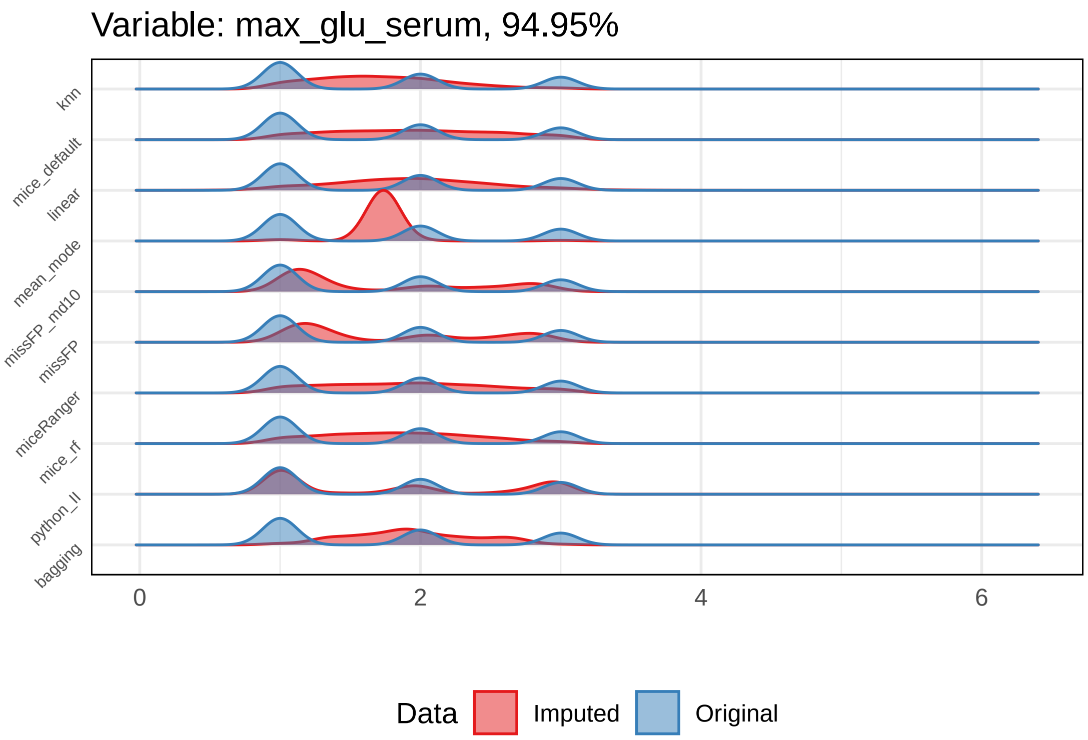

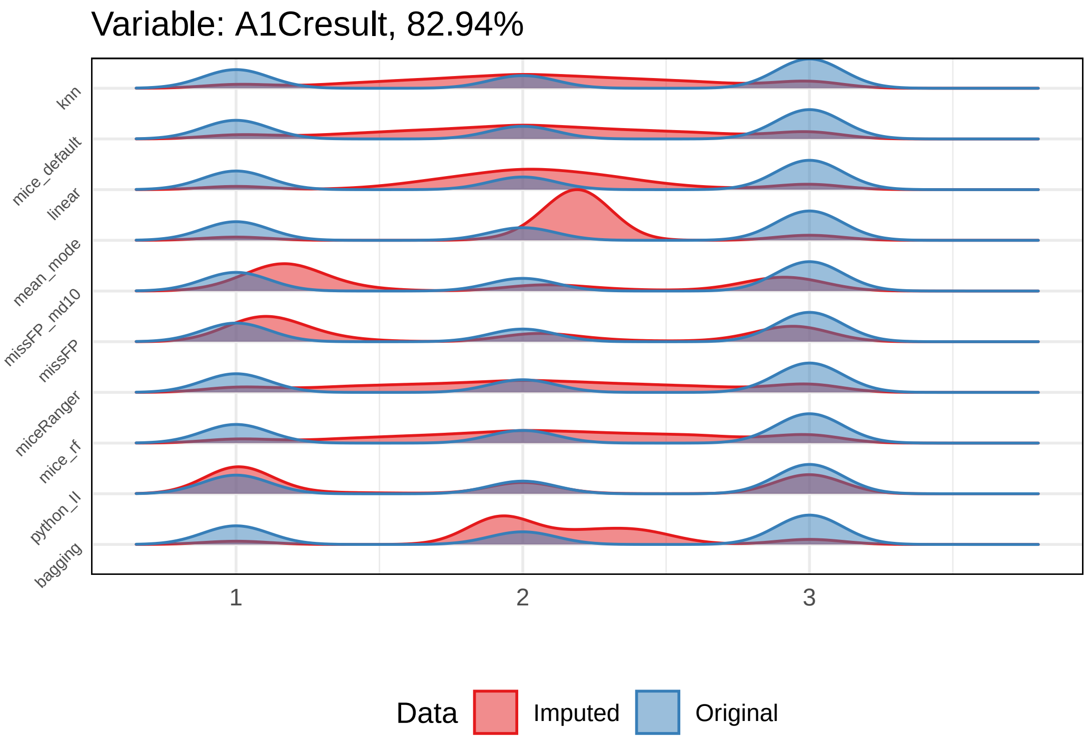

Variable: medical_specialty, 48.78% missing

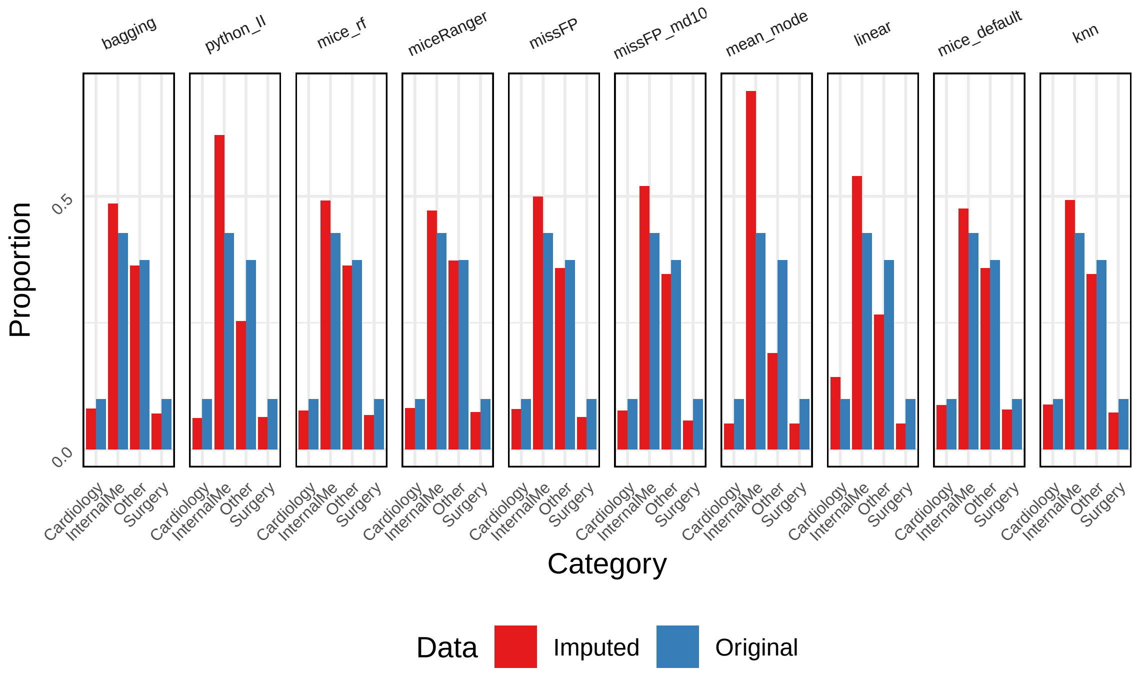

Variable: admission_source_id, 6.73% missing

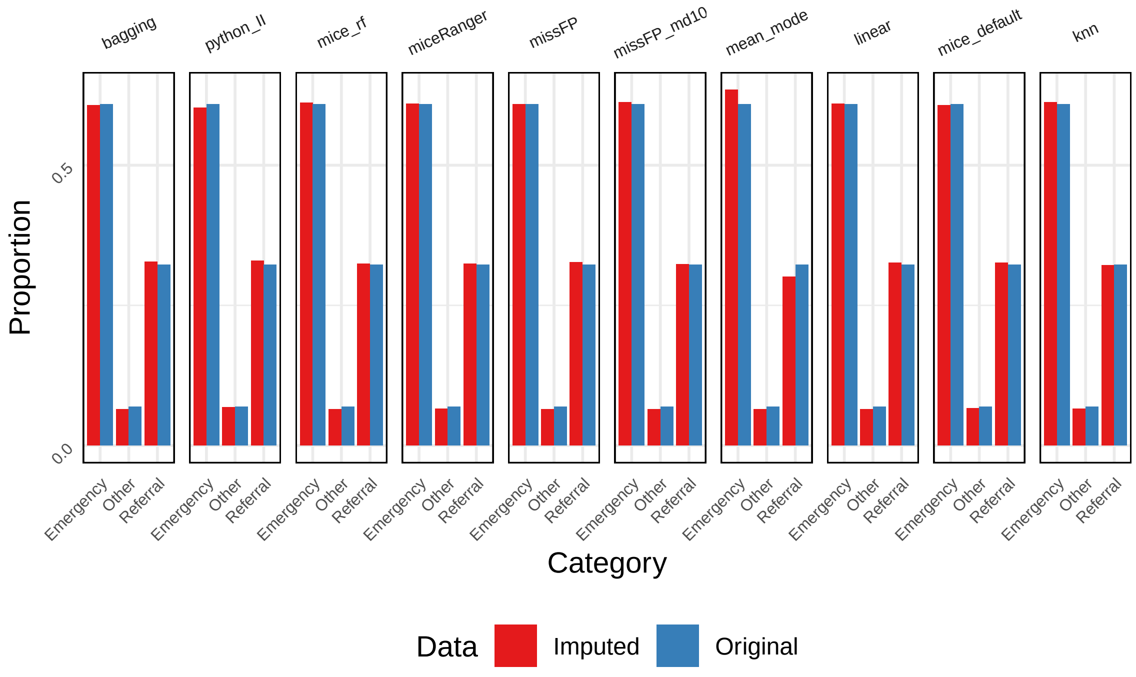

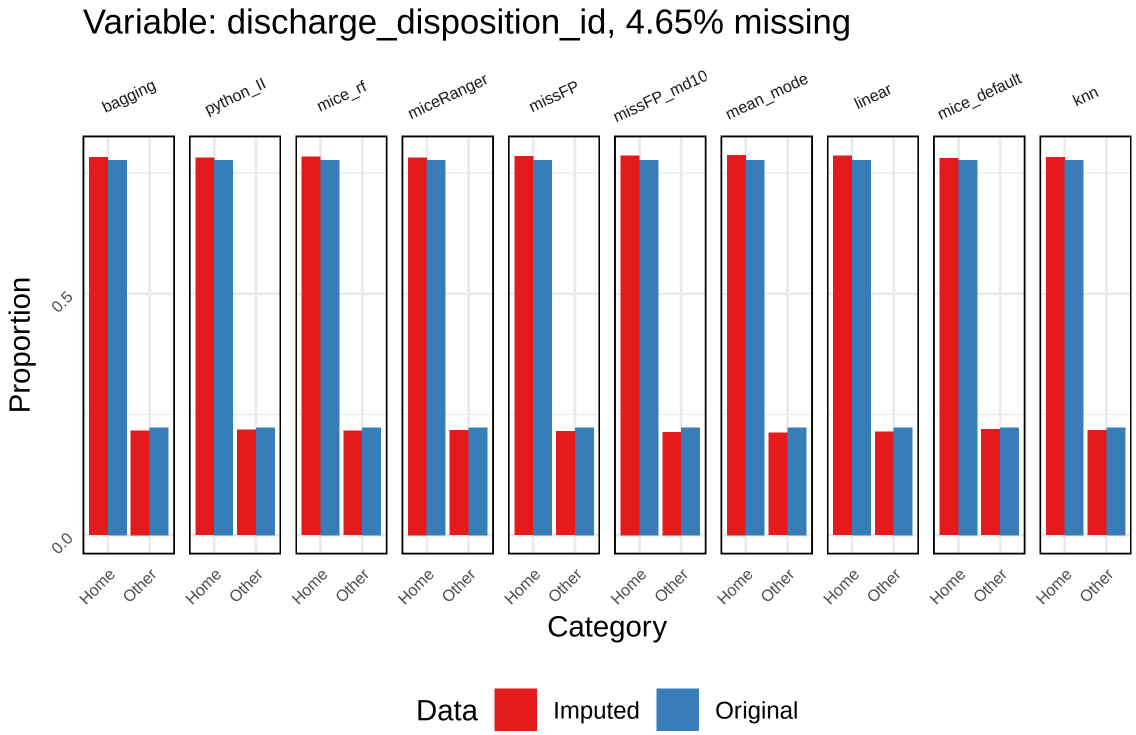

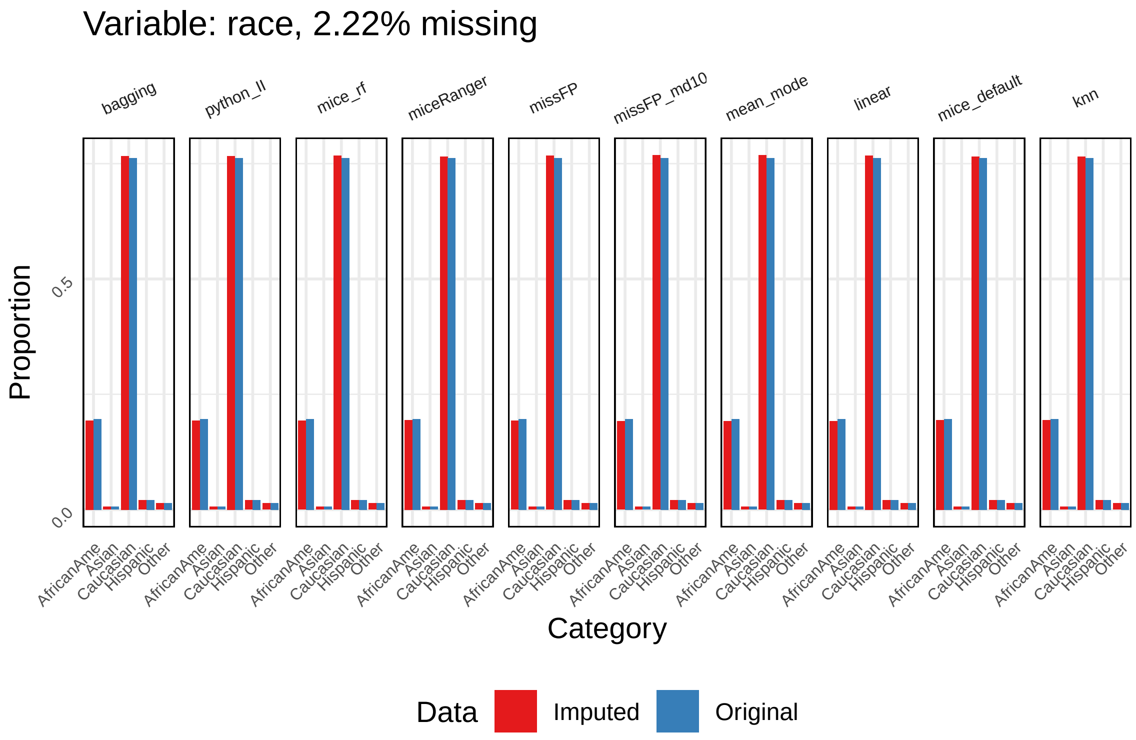

References

Dua, Dheeru, and Casey Graff. 2017. "UCI Machine Learning Repository." University of California, Irvine, School of Information; Computer Sciences. http://archive.ics.uci.edu/ml.

Friedman, Jerome, Trevor Hastie, and Rob Tibshirani. 2010. "Regularization Paths for Generalized Linear Models via Coordinate Descent." *Journal of Statistical Software* 33 (1): 1.

Harrell Jr, Frank E. 2023. *Rms: Regression Modeling Strategies.* https://CRAN.R-project.org/package=rms.

Higgins, Peter. 2021. *Medicaldata: Data Package for Medical Datasets.* https://CRAN.R-project.org/package=medicaldata.

Kuhn, Max. 2008. "Building Predictive Models in r Using the Caret Package." *Journal of Statistical Software, Articles* 28 (5): 1–26. https://doi.org/10.18637/jss.v028.i05.

Le, Trang, makeyourownmaker, and Jason Moore. 2020. *Pmlbr: Interface to the Penn Machine Learning Benchmarks Data Repository.* https://CRAN.R-project.org/package=pmlbr.

Marmot, Michael G, Martin J Shipley, and Geoffrey Rose. 1984. "Inequalities in Death—Specific Explanations of a General Pattern?" *The Lancet* 323 (8384): 1003–6.

Olson, Randal S., William La Cava, Patryk Orzechowski, Ryan J. Urbanowicz, and Jason H. Moore. 2017. "PMLB: A Large Benchmark Suite for Machine Learning Evaluation and Comparison." *BioData Mining* 10 (1): 36. https://doi.org/10.1186/s13040-017-0154-4.

Probst, Philipp, Marvin Wright, and Anne-Laure Boulesteix. 2018. "Hyperparameters and Tuning Strategies for Random Forest." *Wiley Interdisciplinary Reviews: Data Mining and Knowledge Discovery.* https://doi.org/10.1002/widm.1301.

Roberts, I, H Shakur, T Coats, B Hunt, E Balogun, L Barnetson, L Cook, et al. 2013. "The CRASH-2 Trial: A Randomised Controlled Trial and Economic Evaluation of the Effects of Tranexamic Acid on Death, Vascular Occlusive Events and Transfusion Requirement in Bleeding Trauma Patients." *Health Technol Assess* 17 (10): 1–79.

Royston, Patrick, Gareth Ambler, and Willi Sauerbrei. 1999. "The Use of Fractional Polynomials to Model Continuous Risk Variables in Epidemiology." *International Journal of Epidemiology* 28 (5): 964–74.

Sandercock, Peter AG, Maciej Niewada, and Anna Członkowska. 2011. "The International Stroke Trial Database." *Trials* 12 (1): 1–7.

Strack, Beata, Jonathan P DeShazo, Chris Gennings, Juan L Olmo, Sebastian Ventura, Krzysztof J Cios, and John N Clore. 2014. "Impact of HbA1c Measurement on Hospital Readmission Rates: Analysis of 70,000 Clinical Database Patient Records." *BioMed Research International* 2014.

Strobl, Carolin, Anne-Laure Boulesteix, Achim Zeileis, and Torsten Hothorn. 2007. "Bias in Random Forest Variable Importance Measures: Illustrations, Sources and a Solution." *BMC Bioinformatics* 8 (1): 1–21.

Wickham, Hadley. 2016. *Ggplot2: Elegant Graphics for Data Analysis.* Springer-Verlag New York. https://ggplot2.tidyverse.org.

Williams-Johnson, JA, AH McDonald, G Gordon Strachan, and EW Williams. 2010. "Effects of Tranexamic Acid on Death, Vascular Occlusive Events, and Blood Transfusion in Trauma Patients with Significant Haemorrhage (CRASH-2): A Randomised, Placebo-Controlled Trial." *West Indian Med. J*, 612–24.

Wright, Marvin N, and Inke R König. 2019. "Splitting on Categorical Predictors in Random Forests." *PeerJ* 7: e6339.
