# Supplementary material for: missForestPredict—Missing data imputation for prediction settings
Source: PLoS One. 2025 Nov 7;20(11):e0334125. doi: 10.1371/journal.pone.0334125 (PMC12594382; doi:10.1371/journal.pone.0334125)
Supplement: Attachment — Submitted filename: Reponse to reviewers 18sep2025.pdf [file pone.0334125.s004.pdf]

PONE-D-24-33134R2; RESPONSE TO REVIEWERS

REVIEWER #2

I have carefully reviewed the revised manuscript and the corresponding "Response to Reviewers" document. The authors have made substantial and appropriate revisions to address the prior concerns raised by reviewers in earlier rounds. The paper now presents a clear, comprehensive, and well-motivated introduction of the missForestPredict algorithm, which meaningfully extends the widely used missForest imputation method to support prediction-time imputation — a feature of high practical value in numerous applied domains, particularly in clinical decision support, finance, and sensor-based analytics.

Response: Thank you for your valuable input, and for these kind words.

Remaining Suggestions (Minor, Stylistic/Grammatical):

- There are occasional typographical inconsistencies (e.g., spacing around mathematical operators, inconsistent use of "data are" vs. "data is").

Response: We have checked the text and fixed the few issues that we found.

- Some sentences in the Methods section are long and could be split for improved readability, especially in the step-by-step algorithm description and convergence criterion explanation.

Response: We have re-read this section, and we would respectfully like to keep it as is.

- Ensure uniform formatting of symbols and subscripts in equations (e.g., \(y^{(s)}_{obs}\) vs `y(s) obs`).

Response: We have checked the equations in the pdf file and we did not find inconsistencies. All equations appear correct to us.

- Verify consistency in citation formatting according to journal style (e.g., spaces before references in square brackets).

Response: We have checked in-text citation style and corrected a few formatting issues.

- A quick language polishing pass may further enhance reader accessibility, particularly for non-specialist audiences.

Response: Thank you for this comment! We made a small change to the abstract, and considerably enhanced the readability of the introduction and conclusion sections.

JOURNAL COMMENTS

1. the reviewer comments include a recommendation to cite specific previously published works, please review and evaluate these publications to determine whether they are relevant and should be cited. There is no requirement to cite these works unless the editor has indicated otherwise.

Response: unfortunately, in the decision e-mail we did not see any specific works the reviewers want us to cite. We are therefore unsure what this comment relates to.

2. Please review your reference list to ensure that it is complete and correct. If you have cited papers that have been retracted, please include the rationale for doing so in the manuscript text, or remove these references and replace them with relevant current references. Any changes to the reference list should be mentioned in the rebuttal letter that accompanies your revised manuscript. If you need to cite a retracted article, indicate the article's retracted status in the References list and also include a citation and full reference for the retraction notice.

Response: We updated reference 14 (from master thesis to journal publication). All other references appeared correct and up to date to us.
