# Supplementary material for: missForestPredict—Missing data imputation for prediction settings
Source: PLoS One. 2025 Nov 7;20(11):e0334125. doi: 10.1371/journal.pone.0334125 (PMC12594382; doi:10.1371/journal.pone.0334125)
Supplement: Attachment — Submitted filename: Response to Reviewers.docx [file pone.0334125.s002.docx]

**Reviewer comments:**

- Although I realize it's something of an extension of missForest, but it seems like there's a distinct lack of any consideration of the results of the imputation. It's so focused on predictions, although it's only half the process. The manuscript is so complete except for any visualization or analysis of what is imputed. I wouldn't do more than a figure or two. Then readers would have everything in one place.

Answer:

For datasets with simulated missingness we presented the deviation from true values (NMSE) as an analysis of what was imputed. We though lack any such analysis on the datasets with missing values. We added two figures to the main text for these latter datasets comparing distributions of variables before and after the imputation, as well as the following text:

*As the deviation from true values (NMSE) cannot be calculated, because missingness was not simulated but present in these datasets, we present the distributions of continuous variables before and after imputation (Fig 13) and the proportion of values in each category before and after imputation (Fig 14), for categorical variables. We limit the results to the imputations on the first iteration and the top two variables in each dataset with highest missingness. Full results for all variables are available in S1 Appendix. Determining the desired distribution after imputation is difficult without knowing the true values. However, missForestPredict and Iterative Imputer produce values that more closely resemble the original distribution for ordinal variables in the diabetes dataset; these were treated as continuous variables across all imputation methods and predictive models.*

We added distribution comparisons for all variables on datasets with missing values in the supporting info file.

We also adapted the code and added the save_dataset parameter in the config.R to save datasets, for users who want to further inspect the distributions before and after imputation on other datasets.

```
# parameter to save datasets for distribution comparison
# (storage requirements can be high for the large datasets over many iterations)
save_dataset <- TRUE
```

- In figures 2,3,4,6, and 8 the y axis is labeled "value", perhaps that could be renamed "Error" or "NMSE"?

Answer:

The axis label has been changed to “NMSE” for the relevant figures.

- Seems like linear and mean imputation is often times the biggest competitor, could consider moving missForestPredict next to them in the plots, because that's what I was always trying to compare... looking across the rows of dots.

Answer:
We adapted the order of the imputation methods on the x-axis: first, original, then tree-based methods, followed by mean/mode, linear and the others. This order maintains the tree based methods together for ease of comparison, and also places missForestPredict near mean/mode and linear. We applied this order to both NMSE and BSS plots, in the manuscript, the supplementary file and in the shiny app. Excerpt from Fig 3 below for exemplification:

- Line 169, should that p_obs be a subscript "obs"?

Answer:

p_obs and p_miss represent the vector components of the proportion_usable_cases parameter of the main function of the package. We prefer to keep the terminology consistent with the package documentation, as reproduced below:

| proportion_usable_cases | a vector with two components: the first one is a minimum threshold for p_obs and the second one is a maximum threshold for p_miss. Variables for which p_obs is greater than or equal to 1 (by default) will be filtered from the predictor matrix. Variables for which p_miss is lower than or equal to 0 (by default) will be filtered from the predictor matrix. For more details on p_obs and p_miss see the documentation for the prop_usable_cases function. If parameter predictor_matrix is specified, the proportion_usable_cases will be applied to this provided matrix. |
| --- | --- |

We have though slightly adapted the text that introduces this parameter for clarification:

**From**: *The variables used in imputation can also be controlled by using the proportion_usable_cases, which represents the variable-wise proportion of usable cases.*

**To**: *The variables used in imputation can also be controlled by using the proportion_usable_cases parameter, which represents the variable-wise proportion of usable cases and contains two components: p_obs and p_miss.*

- I wonder if a lighter application of noise wouldn't have been better... had a huge effect.

Answer:

Our rationale for adding 12 noise variables is stated in Methods: “Enough noise variables are added to attempt to force RF based methods to do splits on noise variables and study the performance of these methods under noise conditions.” Indeed, the added noise has quite some effect, that differs among imputation methods, and in some scenarios even a surprising effect of “regularization” against “over-imputation”.

There is definitely value in studying the impact of different levels of noise, in terms of number of noise variables for prediction. At the same time we feel the paper has already a lot to communicate, and a deeper exploration of noise settings might be a bit too much for the reader to process.

We realize though that we do not comment enough on the effect of noise in the Discussion section. We have therefore added in Discussion the next sentences:

*Additionally, we investigated the impact of “noise” variables on simulated datasets, by adding twelve noise variables to datasets with four “signal” variables, resulting in a high noise to signal ratio of 3:1. In all scenarios, the addition of noise variables negatively affected on the final performance of the prediction model, which can be due its impact on the imputations, its direct impact on the prediction model, or both. Further research could explore different levels of noise and disentangle the performance decrease attributable to imputation from that caused by the prediction model itself.*

- Nice code!

Thank you!

**Journal requirements:**

1. Please ensure that your manuscript meets PLOS ONE's style requirements, including those for file naming. The PLOS ONE style templates can be found at

<https://journals.plos.org/plosone/s/file?id=wjVg/PLOSOne_formatting_sample_main_body.pdf> and

<https://journals.plos.org/plosone/s/file?id=ba62/PLOSOne_formatting_sample_title_authors_affiliations.pdf>

We have identified the following inconsistencies:

- Figures were referred with Figure x instead of Fig x. This has been corrected.
- We have not used bold type for the tables and figure titles. This has been corrected.
- Tables and figures did not appear directly after the paragraph in which they are first cited. This has been corrected.
- The section Supporting Information was not included in the main manuscript. This has been corrected.
- Corresponding author has been added.

We hope we did not overlook any formatting requirements.

2. We note that the grant information you provided in the ‘Funding Information’ and ‘Financial Disclosure’ sections do not match.

 When you resubmit, please ensure that you provide the correct grant numbers for the awards you received for your study in the ‘Funding Information’ section.

Answer:

The provided text matched exactly in the initial submission:

Financial disclosure:

This research was funded by KU Leuven funds (grant application C24M/20/064). The funding sources had no role in the design of the study, the collection, analysis, and interpretation of data, or in writing the manuscript.

Funding information:

This research was funded by KU Leuven funds (grant application C24M/20/064). The funding sources had no role in the design of the study, the collection, analysis, and interpretation of data, or in writing the manuscript.

 We will resubmit the same text, kindly point us to precise discrepancies, if any remain.

3. Please include captions for your Supporting Information files at the end of your manuscript, and update any in-text citations to match accordingly. Please see our Supporting Information guidelines for more information: <http://journals.plos.org/plosone/s/supporting-information>.

Answer:

Supporting Information has been referred at the end of the manuscript.

4. Please review your reference list to ensure that it is complete and correct. If you have cited papers that have been retracted, please include the rationale for doing so in the manuscript text, or remove these references and replace them with relevant current references. Any changes to the reference list should be mentioned in the rebuttal letter that accompanies your revised manuscript. If you need to cite a retracted article, indicate the article’s retracted status in the References list and also include a citation and full reference for the retraction notice.

Answer:

We have identified one reference that no longer exists as an arXiv preprint, but was meanwhile published in Statistical Papers, we have adapted the reference accordingly:

FROM:

Josse J, Prost N, Scornet E, Varoquaux G. On the consistency of supervised learning with missing values. arXiv preprint arXiv:190206931. 2019.

TO:

Josse J, Chen JM, Prost N, Varoquaux G, Scornet E. On the consistency of supervised learning with missing values. Statistical Papers. 2024;65: 5447–5479.
