# Supplementary material for: missForestPredict—Missing data imputation for prediction settings
Source: PLoS One. 2025 Nov 7;20(11):e0334125. doi: 10.1371/journal.pone.0334125 (PMC12594382; doi:10.1371/journal.pone.0334125)
Supplement: Attachment — Submitted filename: Response_to_Reviewers_auresp_2.docx [file pone.0334125.s003.docx]

**Reviewers' comments:**

Reviewer #1: Thanks for fully addressing the concerns. It’s a nice paper, good work. I’m sure it will be useful.

**Response**: We thank the reviewer for taking the time to carefully read and revise the manuscript, as well as the code. We appreciate the effort!

Reviewer #2: Manuscript ID: PONE-D-24-33134R1
Title: missForestPredict – Missing data imputation for prediction settings

Dear Authors,

Thank you for your revised submission. After a careful and detailed review of your manuscript, I would like to share several concerns and points that require further attention and clarification. While your manuscript addresses an important topic, there are a number of methodological and conceptual issues that, in my opinion, weaken the current version and limit its clarity and practical utility.

1. Limited Justification for Convergence Criterion
Your proposed use of NMSE as a unified convergence criterion is not sufficiently justified. Specifically, applying the same NMSE thresholding logic to both continuous and categorical variables (via Brier Skill Score transformations) lacks nuance. The metric’s sensitivity to data imbalance—especially for categorical variables—is not discussed, and no rationale is provided for why the default convergence behavior (e.g., stopping when NMSE increases) is ideal across scenarios. This calls into question the robustness of the convergence mechanism.

Recommendation: Provide a more detailed rationale for choosing NMSE across variable types, include sensitivity analyses, and justify the default convergence thresholds through empirical or theoretical reasoning.

**Response**: Iterative imputation algorithms use two ways of stopping (1) at convergence (original missForest, IterativeImputer) or (2) after a fixed number of iterations (mice, miceRanger); missForestPredict supports both (see package documentation).

Our default convergence metric (NMSE), further explored in the package vignette (<https://cran.r-project.org/web/packages/missForestPredict/vignettes/missForestPredict_convergence_criteria_and_error_monitoring.html>) unifies the original missForest convergence criterion for continuous and categorical variables and can be customized by:

- User control over parameters var_weights, oob_error; the package vignette demonstrates the change in number of iterations as a result of change in the weights
- Visual inspection of errors and bypass convergence by setting maxiter to a fixed number of iterations

We did not undertake a full theoretical or experimental study of optimal convergence for different scenarios, different variable types, or variables distributions; neither do other imputation methods implementations.

However, while developing the package, we conducted some back-of-the-envelope comparisons on iris and diamonds datasets. Using simulated MCAR missingness, we compared 1 iteration vs. convergence vs. 10 iterations in terms of variable-wise errors: MSE (mean square error) for continuous variables and PFC (proportion falsely classified or misclassification error rate) for categorical variables. Convergence proved best in all scenarios. We include excerpts below for the diamonds dataset. We have also conducted other such minimal ‘sensitivity analyses’ for the initialization scheme and comparison of the new convergence criterion the old convergence criterion of the missForest algorithm. We have not included these in the study, and nor as vignettes, as we considered them technical testing during development phase. While these would not constitute strong evidence of the superiority of default package choices (convergence, initialization), the best evidence that the default convergence criterion is adequate represents the results of the current study (covering 48 simulated scenarios + 8 real datasets), which prove the good performance of missForestPredict (which has been run with the default convergence criterion in all studied scenarios).

In the Discussion section we ask a very bold question. Considering the very good performance of bagging (despite being the most memory intensive), we wonder if iterative algorithms (missForestPredict and others) are not overdoing by iterating (at least for prediction settings)? We leave space to other researchers to study the behaviors of different algorithms under different number of iterations or until convergence.

During our internal discussions, we also asked ourselves the question: where in the feature space does the algorithm converge? Does the state of the feature space at convergence have any interpretation? How does this vary for different convergence criteria? While this is a beautiful theoretical question, and it depends not only on the convergence criterion but also on other algorithmic implementation details (e.g. initialization scheme, order of variables in imputation, variables imputed), answering such questions has virtually no practical value. With the current knowledge, we view convergence criteria in iterative imputation algorithms mainly as a practical means to prevent algorithms from running too long or too few.

With all this being said, we have added the below sentences to the manuscript, without making any strong claims about the robustness of the convergence criterion. We consider that further justifying the convergence criterion would not bring much added value.

2. Scalability and Memory Use Concerns
The method stores imputation models for each variable and iteration, but the implications for scalability in high-dimensional datasets are not discussed. It is unclear how missForestPredict performs when applied to datasets with hundreds or thousands of variables (e.g., genomic or sensor data).

Recommendation: Include runtime and memory usage benchmarks on at least one high-dimensional dataset, or explicitly clarify the limitations and trade-offs of the current implementation in such settings.

**Response**: Our inclusion criteria for datasets studied imposed 10000 observations at least. While searching public datasets, no high-dimensional (that would mean p > 10000) or sensor data fell within our criteria. We must add that searching for suitable public datasets for the study constituted a time consuming task.

In Discussion we already admit that our inclusion criteria might have been too stringent.

We have further added the number of variables with missing values as an additional limitation. Researchers with experience (and access to) genomic and sensor data can further study realistic use cases and make further practical recommendations.

For information, we have successfully used the algorithm on a large dataset with 66 variables with missing values in one of our further studies: <https://link.springer.com/article/10.1186/s12879-025-10854-1>

3. Lack of Error Analysis
Although your results section reports predictive and imputation performance across scenarios, it fails to offer any qualitative or case-specific analysis of failures, misclassifications, or patterns of poor performance (e.g., in MNAR or MAR scenarios). This weakens interpretability and hinders practical guidance for users.

Recommendation: Include an error analysis section with case studies or diagnostic plots highlighting when and why the method fails or overfits (i.e., "over-imputes").

**Response**: For all scenarios with simulated missingness we present the NMSE errors (deviations from true values) on test sets. We also allocate a full paragraph in each Results subchapter to present these results, MAR and MNAR scenarios included. We need to point out that missForestPredict provides among best results in all scenarios, with the most noticeable difference in low correlation settings, where we see a difference between missForestPredict with deep vs. shallow trees.

In the Discussion section, we further comment on these results, putting them in a broader context. We comment on the differences between missForestPredict with deep vs. shallow trees, and explain these difference through the lens of the convergence criterion and the internal mechanism of random forests. We also provide recommendations for identifying low vs. high imputability settings for avoiding over-imputation. We consider the information provided in the Results and Discussion sections sufficient and we do not feel the need to create a separate Error analysis section, as suggested in the reviewer’s recommendation. The scenario in which missForestPredict fails / overfits is fully discussed.

We must also point out that kNN, miceRanger and Iterative Imputer tend to over-impute. Mice also underperforms on some real datasets. We though do not explain the lower performance of each imputation method in each scenario, as our focus was to compare missForestPredict to other algorithms, and not to study in detail the properties and behavior of each algorithm.

4. Insufficient Comparison to Contemporary Methods
While the study compares to several common imputation tools (e.g., mice, miceRanger, IterativeImputer), there is no mention or evaluation of modern deep learning or transformer-based imputation frameworks, or techniques that model missingness explicitly as informative.

Recommendation: Acknowledge the limitations of not including such baselines, or ideally, include one or two such recent methods for a fairer benchmark.

**Response**: While we acknowledge the growing interest in deep learning methods in the current research landscape, our study deliberately focused on RF-based imputation methods, complemented by popular imputation methods available in generic libraries like caret, tidymodels and scikit-learn. The rationale for imputation methods selection is clearly described in the Methods section, Imputation methods subsection (excerpt below). In the Supplementary materials, we include all methods assessed for inclusion in the comparison, and the criteria on which they were evaluated for inclusion. This clearly justifies our scope and, in this light, we do not consider the exclusion of deep learning methods as a limitation, nor do we consider that including deep learning would result in a “fairer benchmark.”

In our Discussion section we briefly mention as limitation the limited number of algorithms compared, as well as the exclusion of model embedded imputation schemed. We consider this sufficient for the scope of the current study. Although future studies could compare deep learning and RF-based imputation methods, we chose not to include this as “future directions” in the Discussion section. Our Discussion section is already extensive, proposing research directions that directly emerge from our study’s findings. We are not aiming to set broader directions for future work.

5. Ambiguity in Parameter Recommendations
Several user-controllable parameters (e.g., number of trees, max depth, p_obs, p_miss) are introduced but not thoroughly explored or explained in terms of their practical implications or impact on performance.

Recommendation: Add guidance, either empirically or heuristically derived, on how to set these parameters in various scenarios. This could be supported by a sensitivity analysis.

**Response**: Out of the user controllable parameters (either missForestPredict parameters or ranger parameters), we have fully explored max_depth in all scenarios and commented on its effect in terms of overall imputation performance as well as of memory requirements. We have focused on this parameter specifically because of practical implications for large datasets, as most prediction problems are solved on large datasets.

We have set the number of trees to a fixed value for all RF methods to compare methods on par, we argument this choice and we believe that this is the best choice for the study’s goal. We though provide an example of altering the number of trees in the package vignette “Using the missForestPredict package”. In the same vignette we provide examples for custom initialization and proportion_usable_cases (p_obs, p_miss).

We consider the exploration of user modifiable parameters included in the current study and in the package vignettes sufficient.

6. Poorly Structured Presentation of Results
The results section is overly long, dense, and at times repetitive. The placement of figures, especially for simulated data, precedes the more practically relevant real-data analysis. Important findings are buried in lengthy narrative without adequate summary or interpretation.

Recommendation: Restructure the results to highlight practical findings more clearly, streamline the narrative, and consider summarizing key patterns in performance using tables or concise bullet-point summaries.

**Response**: We agree that the results section is rather lengthy. To facilitate reading, the first paragraph of each subsection represents a summary paragraph, for example:

We also made a conscious effort to avoid redundancy, i.e. whenever the results are similar to the previous section we state that, and highlight only the differences, for example:

We deliberately chose not to aggregate or summarize results across scenarios, datasets, or models, as doing so would dilute findings that are relevant only in particular settings (e.g. only for low correlation, or only for tree-based prediction models).

We studied 48 simulates scenarios, 8 real datasets, over 4 prediction models and 10 imputation methods, which represents a rather large scope compared to most other studies to date. To preserve transparency and avoid selection bias, we opted for a detailed and direct presentation of the raw results, rather than filtering or condensing them. This approach mirrors the structure of the accompanying Shiny app, which provides users with additional metrics (e.g., AUROC, calibration metrics), as well as the option to explore results in table format (<https://sibip.shinyapps.io/Results_imputation_methods/>). The app is intended for readers that want to drill deeper into results.

The objective of the study is to compare missForestPredict to other imputation methods. In this light, the goal of the Results section is not to highlight practical findings, but to report how methods compare. We intentionally left interpretation of key findings, as well as practical recommendations that result from these, for the Discussion section (paragraphs 3 to 7), where we discuss the main findings in a broader practical context. The readers interested in the summary of main findings can skip directly to the Discussion section.

7. Reproducibility and Workflow Transparency
While code is provided, there is no clear description of the workflow pipeline for reproducing the experiments (especially test-time imputation), nor a discussion on how the models generalize across datasets with varying missing data mechanisms.

Recommendation: Include a clear, schematic overview of the full workflow (perhaps in an appendix or figure), and discuss limitations in generalizing trained imputation models to datasets with differing missingness patterns.

**Response**: Table 7, “Comparison procedure steps”, details step by step the workflow pipeline. The last four steps clearly describe the actions on the test set, including test-time imputation.

Additionally, the code is documented on the github repository (<https://github.com/sibipx/comparison_imputation_methods>). We also provide clear step-by-step instructions on how to reproduce the experiments on a new dataset (or on the shared datasets). We emphasized the reusability of the code in the manuscript.

We have difficulty understanding what the reviewer means by “how the models generalize across datasets with varying missing data mechanisms”. We first guess that “models” indicates imputation methods and not prediction models. We can interpret “varying missing data mechanisms” as different missing data mechanisms, which we studied quite extensively. Or varying the missing data mechanism between the training and the test set (dataset shift on missing data mechanism). The latter is an interesting research question, which is far off-topic from what we studied. We have already made clear the use case of interest which defines the scope of our study.

We also have difficulty understanding why workflow and code transparency would be grouped with this request for extension of missing data mechanism shift from train to test. An extension that we do not consider necessary, but can be further studied by other research groups.

In summary, although the manuscript presents an extension to an existing method, it currently lacks the depth and rigor necessary to fully support its contributions and claims. A substantial revision addressing the above concerns is required before this work can be considered for publication.

**Response**:

We respectfully disagree with the reviewer’s general statement that the manuscript “lacks the depth and rigor necessary to fully support its contributions and claims.” We believe this judgment overstates the issues raised and several rationales for analyses appear to have been overlooked or misinterpreted.

The contributions of the current paper are:

1) an R package adapting the missForest algorithm to prediction settings when data are missing at prediction time; it is fast, user friendly, functionality rich and competitive in prediction settings.
2) an extensive comparison of 10 imputation methods (RF-based and widely used alternatives in R/Python) combined with 4 prediction models, across 48 simulated scenarios and 8 real datasets
3) practical recommendation for identifying low vs. high imputability settings; findings on how different imputation methods interact with prediction models; practical advice for computation time and memory utilization when imputing large datasets.

Based on our findings, we claim that missForestPredict is suitable for prediction settings when data are expected to be missing at prediction time.

These contributions and claims are backed by rigorous methodology: transparent experimental design, publicly available and well-documented code and data, and one of the most comprehensive evaluation scopes on this topic to date.

We have not:

- Justified the convergence criterion in depth – but it is user configurable (remark 1)
- Included high dimensional data (genomic and senor data) – but we included as much relevant data for prediction tasks we could find publicly (remark 2)
- Compared to deep learning imputation methods – but we clearly explain the rationale for imputation methods inclusion in the study (remark 4)

We have:

- Provided sufficient error analysis, with explanation of error situations for missForestPredict (remark 3)
- Explored the package parameters either in the study or in the package vignettes (remark 5)
- Summarized the key results and practical insights in the Discussion section (remark 6)
- Provided clear workflow information, as well as open and well documented code, making our work both reproducible and reusable on new datasets (remark 7)

While our study does not cover all possible scenarios (e.g., genomic data, deep learning imputation, all possible parameter combinations), which no single paper can, we have made deliberate and justified methodological choices. While we have not been exhaustive across every possible methodological variation (and we encourage other researchers to take the work further by embracing open science and software principles), our work is more extensive than most studies on the same topic to date. The open code is well structured, well documented and easy to reuse.
